# Update on Methodologies Available for Ciguatoxin Determination: Perspectives to Confront the Onset of Ciguatera Fish Poisoning in Europe [1]

**DOI:** 10.3390/md8061838

**Published:** 2010-06-14

**Authors:** Amandine Caillaud, Pablo de la Iglesia, H. Taiana Darius, Serge Pauillac, Katerina Aligizaki, Santiago Fraga, Mireille Chinain, Jorge Diogène

**Affiliations:** 1 IRTA, Ctra. Poble Nou, Km 5,5. 43540 Sant Carles de la Ràpita, Spain; E-Mails: amandine.caillaud@irta.es (A.C.); pablo.delaiglesia@irta.es (P.I.); 2 Laboratoire des micro-algues toxiques, Institut Louis Malardé, BP30, 98713 Papeete Tahiti, French Polynesia; E-Mails: tdarius@ilm.pf (H.T.D.); mchinain@ilm.pf (M.C.); 3 Institut Pasteur, 25-28 rue du docteur Roux, 75 015 Paris, France; E-Mail: serge.pauillac@pasteur.fr (S.P.); 4 Department of Botany, School of Biology, Faculty of Sciences, Aristotle University, 54 124 Thessaloniki, Greece; E-Mail: aligiza@bio.auth.gr (K.A.); 5 Instituto Español de Oceanografía, Subida a Radio Faro, 50, 36390 Vigo, Spain; E-Mail: santi.fraga@vi.ieo.es (S.F.)

**Keywords:** ciguatera, CFP, ciguatoxin, detection methods, Gambierdiscus, methods, Europe

## Abstract

Ciguatera fish poisoning (CFP) occurs mainly when humans ingest finfish contaminated with ciguatoxins (CTXs). The complexity and variability of such toxins have made it difficult to develop reliable methods to routinely monitor CFP with specificity and sensitivity. This review aims to describe the methodologies available for CTX detection, including those based on the toxicological, biochemical, chemical, and pharmaceutical properties of CTXs. Selecting any of these methodological approaches for routine monitoring of ciguatera may be dependent upon the applicability of the method. However, identifying a reference validation method for CTXs is a critical and urgent issue, and is dependent upon the availability of certified CTX standards and the coordinated action of laboratories. Reports of CFP cases in European hospitals have been described in several countries, and are mostly due to travel to CFP endemic areas. Additionally, the recent detection of the CTX-producing tropical genus *Gambierdiscus* in the eastern Atlantic Ocean of the northern hemisphere and in the Mediterranean Sea, as well as the confirmation of CFP in the Canary Islands and possibly in Madeira, constitute other reasons to study the onset of CFP in Europe [1]. The question of the possible contribution of climate change to the distribution of toxin-producing microalgae and ciguateric fish is raised. The impact of ciguatera onset on European Union (EU) policies will be discussed with respect to EU regulations on marine toxins in seafood. Critical analysis and availability of methodologies for CTX determination is required for a rapid response to suspected CFP cases and to conduct sound CFP risk analysis.

## 1. Introduction

Ciguatera Fish Poisoning (CFP) is a human intoxication caused by the consumption of fish that contain ciguatoxins (CTXs) [2]. The geographical distribution of CFP occurrence is mainly limited to tropical and subtropical areas. CFP is found endemically within the western Atlantic Ocean (including the Caribbean Sea), in the Indian Ocean, and in the Pacific [3]. Symptoms of the intoxication are used to diagnose and distinguish CFP from other seafood intoxications. However, the confirmation of cases of CFP relies upon the detection of CTXs in the remaining meal or within the plasma of patients [4]. Therefore, it is important to have adequate CTX quantification methods to diagnose CFP cases and moreover, to prevent intoxications through the analysis of consumable fish.

CTXs are secondary metabolites produced by the marine benthic dinoflagellate of the genus *Gambierdiscus*. Numerous congeners of CTXs have been identified according to differences in their molecular structure. CTXs from distinct geographical origins (e.g., Pacific, Caribbean, and Indian CTXs) have been described. Some CTXs are transmitted through metabolism in the food chain. The high toxicity and diversity of CTXs, along with their presence at trace levels in fish tissue, make their reliable detection and quantification in fish flesh difficult. Numerous methodologies based upon different approaches (e.g., toxicological symptoms, antibody recognition, mass spectrometry, *etc.*) have been developed for CTX recognition. Their applicability not only depends on the sensitivity and specificity of the method (ideally, the method is sensitive to all CTX congeners), but also relies on effective extraction and clean-up procedures, which are necessary to prepare samples for analysis. In the present review, a selection of protocols for sample preparation prior to the application of different methodologies of CTX determination is described. A revision of the different approaches available for CTX determination, *i.e.*, *in vivo* mouse bioassay (MBA), *in vitro* cell based assays (CBA), competitive receptor binding assay (RBA), immunological analysis, and physicochemical analysis (HPLC), are further described. The applications of these assays as routine screening tools of CTXs in fish and *Gambierdiscus* spp. samples are also discussed.

The interest for CFP in Europe has been increasing during the last years as CFP has been reported in the hospitals of Europe (e.g., France, Spain, the Netherlands, Germany, and Italy), usually in people who consumed imported ciguateric fish or who travelled to CFP areas [5–8]. Recent reports indicate that CFP is present in areas of Africa close to Europe. In 2004, CFP was first confirmed after consumption of flesh from fish caught in the Canary Islands [9]. The presence of CTXs was also suspected in fish from Madeira in 2007 and 2008 [10]. Additionally, identification of species of dinoflagellates of the genus *Gambierdiscus*, which are potential CTX producers, has been recorded in the Mediterranean Sea since 2003 [11–14], and for the first time in the waters of the Canary Islands in 2004 [15].

Extensive reviews on CFP provide comprehensive analyses regarding the importance of CFP and the different factors that influence it [2,3,16–24]. The present review gives priority to two subjects. First, as introduced before, there is a review of CTX determination methods, so as to update readers of the available strategies that may improve in the future, and also to standardize and prioritize the appropriate methods for CFP management worldwide. Second, in light of the onset of CFP in Europe, the paper addresses the importance of the present evidence concerning the onset and evaluates possible measures to confront this potential crisis. In a context where the importance of CFP may be recognized world-wide, and considering the possible increase of CFP cases in traditionally non-endemic areas in Europe and elsewhere, it becomes clear that CFP risk analysis has to be improved to reduce CFP impact.

## 2. General Considerations about CFP

### 2.1. Origin of CFP and toxic fish

The food chain hypothesis, which suggests that the transfer of toxins along the food web is an explanation for CFP movement, was first proposed by Mills. Mills postulated a role for the phytoplankton in toxin synthesis [25]. This implication was later supported by Randall [26]. Further confirmation arrived by isolation of a toxic marine benthic dinoflagellate, posteriorly described as *Gambierdiscus toxicus*; it was seen as a likely source of CTXs [27]. Adachi and Fukuyo [28] confirmed that wild and cultured *G. toxicus* produce precursors of CTXs (known as gambiertoxins) [29]. Transfer of CTXs along the food web was suggested to start with dinoflagellates before moving on to herbivorous grazing fish, and then to the carnivorous fish that fed upon the grazers [30]. Some of these toxins accumulate in the fish tissue and can be metabolized into the different forms (e.g., CTX-1B and 51-hydroxyCTX-3C) from original CTX structures (e.g., CTX-3C and CTX-4B) that are ultimately responsible for human intoxication.

Both herbivorous and carnivorous fish from tropical areas can be toxic. More than 400 fish species have been reported to be potentially ciguateric [31]. In several areas, it has been possible to recognize the most hazardous fish species. For example, in some parts of the Caribbean Sea, barracuda (*Sphyraena* spp.) are considered extremely hazardous CFP vectors. Identifying fish species at risk is certainly useful for fisheries management and for the establishment of public health regulations. However it is not possible to generalize and establish a list of hazardous species worldwide; there is significant variation in toxin content between individuals within the same species and between geographic areas. A general consideration in a given CFP endemic area is that within the same species, young fish are less hazardous than the older and larger ones. A shorter lifespan means a shorter time span available for toxin accumulation in fish tissue. Oshiro *et al.* [32] stated that the toxicity of larger fish is higher than that of smaller ones. Additionally, carnivorous fish, and those situated at higher trophic levels within the food webs, can be considered riskier than those situated at lower levels (herbivorous). However, these notions have been questioned by Darius *et al.* [33], who described no clear relationship between the size and weight of fish in samples from French Polynesia and CFP levels. The group also observed that herbivorous fish were key vectors of CFP [34]. CFP toxins accumulate mainly in the viscera of fish, but are also present in the muscle and other parts of the body [31,35]. Furthermore, it is important to distinguish fish involved in CFP from fish involved in other forms of poisoning (e.g., ichtyosarcotoxaemias) for which the causative agents and symptoms are different. This is the case for pufferfish poisoning (caused by tetrodotoxin), scombromoid fish poisoning (histamine), clupeoid poisoning, elasmobranch poisoning, mercury fish poisoning [36], or caulerpa poisoning [37].

### 2.2. Epidemiology and symptomatology

The epidemiology of CFP is difficult to assess accurately since it is widely recognized that CFP cases are underreported [38]. Still, numerous epidemiological data exists on CFP [39–43]. It is important to state that significant differences in symptoms may exist between patients. Differences also exist among symptoms presented by patients from distinct geographical areas [44]. These differences are most probably due to structure-activity and pharmacokinetic variations encountered between the different CTX congeners [45,46]. Even within areas, the risk of CFP is not homogenous, and the incidence of CFP varies.

About 400 million people live in areas where CFP is present. In 1998, the worldwide incidence of ciguatera was estimated to affect annually between 50,000 and 500,000 individuals [19,47]. CFP is rarely fatal with fatalities estimated to be <0.1%; however, fatalities may be higher in the Indian Ocean [48]. A survey from 2000 to 2008 in French Polynesia reported a mean incidence rate of around 23 ± 6.5 cases per 10,000 per year [34]. The highest incidence rate was recorded at 464 cases per 10,000 in 2002 in Raivavae Island [34]. Among the 47 fish species implicated as responsible in French Polynesia, most were carnivorous (68%), followed by herbivorous (21.3%), and omnivorous (10.3%) species [43]. In New Caledonia, a survey among the population showed that 70% of the population had experienced CFP [49]. A recent survey conducted in 2005 in Nouméa suggested that the CFP prevalence rate even increased from 25% in 1992 to 37.6% in 2005 [50]. The fish species most incriminated in CFP cases were carnivorous (85%) [51], followed by herbivorous species. Additionally, four cases were related to shellfish consumption [50].

Clinical manifestations appear 2–30 h after consumption of ciguateric fish. The symptomatology of CFP is quite complex; there are many toxicological manifestations at different levels. The symptoms of CFP involve general (e.g., weakness, joint pains, back stiffness, myalgia, headache, chills, faintness, dizziness, oliguria, and itching), digestive (e.g., nausea, vomiting, diarrhea, abdominal pain, cramps, and dehydration), cardiovascular (e.g., low arterial pressure, irregular heartbeat, and bradycardia), and neurological (e.g., dysaesthesia, temperature reversal, paresthesia, superficial hyperesthesia, mydriasis, and absence of the patellar and achillean reflex) pathologies [26,39,52]. Some neurological symptoms are characteristic of CFP, such as dysaesthesia (reversal of cold and hot sensation/hypersensitivity to cold) and paresthesia (lack of sensitivity in the extremities). CFP neurological effects can also last for months, and occasionally, years [38,53]. Alcohol consumption, tobacco smoking, and fish consumption have been shown to promote or reactivate symptoms [53,54]. According to numerous case studies and reports, the intravenous injection of mannitol (1 g kg^−1^ over 30 min) early after CFP seems to be the most efficient therapy for CFP [36,55,56]. Other palliative treatments for CFP intoxication have been implemented with some success, such as a a mixture of vitamins (C and B) and calcium gluconate in glucose solution [57], and other treatments oriented towards the mitigation or reversion of specific symptoms [36,58,59]. Folk remedies have been traditionally used to prevent or treat CFP-associated symptoms [60]. For instance, in a study by Boydron-Le Garrec *et al.* [61], 27 plant extracts from 31 plants tested showed *in vitro* protective effects against CTX action, thus leading to the possible isolation of new bioactive compounds that can treat CFP [62]. Recently, brevenal, a polyether compound produced by the dinoflagellate *Karenia brevis*, was described as a potent inhibitor of CTX-induced neurotoxic effects; acting through the inhibition of CTX-induced catecholamine secretion [63]. All of these compounds may be considered as new candidates for CFP treatment [62–64].

### 2.3. Organisms producing CTXs

*Gambierdiscus toxicus* has been considered the main species responsible for the production of CTXs. This marine dinoflagellate usually grows in tropical and subtropical waters as an epiphyte on macroalgae in coral reefs, mangroove systems, and on artificial surfaces [65] or sand [66]. Five additional *Gambierdiscus* species were later described: *G. belizeanus* [66], *G. yasumotoi* [67], *G. polynesiensis*, *G. pacificus*, and *G. australes* [68]. Production of toxins by some of these species has been described [67–70], but their direct implication in CFP outbreaks has never been confirmed. However, cases attributed to *G. toxicus* could have been caused by other *Gambierdiscus* species.

These studies, along with recent works [71,72], underline the high degree of complexity in *Gambierdiscus* taxonomy. Due to uncertainties regarding the identification of *Gambierdiscus* species, strongly hampered studies on the ecology of this dinoflagellate, an extensive revision of the six already described species was further conducted by Litaker *et al.* [73]. The revision was based upon morphological and phylogenic analysis, and led to the description of four new species, *i.e.*, *G. caribaeus*, *G. carolinianus*, *G. carpenteri*, and *G. ruetzleri* [73]. It was also proposed that the original species, *G. toxicus*, of Adachi and Fukuyo [28], may in fact include multiple species [72,73]. However, no toxicological data is currently available regarding these four new species.

Ecological and physiological studies have been conducted on both wild and cultured *Gambierdiscus* spp. in order to elucidate the factors influencing the occurrence of toxic *Gambierdiscus* blooms. The existence of genetically determined species producing CTXs was first proposed by Holmes *et al.* [74], and is supported by recent data that identified three CTX super-producing clones of *G. polynesiensis* among 20 *Gambierdiscus* clones tested [69]. The role played by environmental conditions in favoring the growth of toxic clones *in natura* should also be considered [75]. The exact nature of factors influencing the toxicity and abundance of *Gambierdiscus* spp. remains unclear. However, light intensity, salinity, water temperature, nutrients, growth stage, and the presence of bacteria have been shown to influence the growth and toxicity of *Gambierdiscus* spp. [76–79].

Other toxin-producing dinoflagellates coexist with *Gambierdiscus* spp. in ciguatera endemic regions, such as species of the genera *Prorocentrum*, *Ostreopsis*, *Coolia*, and *Amphidinium*. Their involvement in CFP is debatable; these species do not produce CTXs, but do produce other toxins. The cyanobacteria of the genus *Hydrocoleum* Kützing was recently proposed as a new additional source of CFP based upon the occurrence of unclassical CFP outbreaks after the consumption of giant clams and molluscivorous fish [80]. However, these poisoning events were recently suggested to be linked with the accumulation of the neurotoxin homoanatoxin-a [81,82].

Many bioactive compounds have been isolated from *G. toxicus, i.e.*, maitotoxins (MTXs) [27,83], gambierols [84], and gambieric acid [85,86]. MTX increases intracellular calcium [87] and is considered one of the most potent among marine biotoxins when injected intra-peritoneally (i.p.) in mice, with an LD50 (i.p. 24 h.) of 50 ng.kg^−1^ [88]. However, MTX has no proven role in human intoxication due to its low capacity for accumulation in fish flesh and low oral potency [88]. Gambierol was described as a potent potassium voltage-gated channel blocker [89] and was suspected to participate in the symptoms of CFP [90,91]. Gambieric acids were isolated from the culture medium of *G. toxicus* and were described as potent antifungal polyether compounds [86].

### 2.4. Chemical structure of CTXs and geographical variability

Ciguatoxins are lipophilic polyethers isolated from fish and *Gambierdiscus* spp. cell extracts (wild samples and cultures). Ciguatoxins are odorless, colorless, devoid of heteroatoms other than oxygen, and bear few conjugated bonds. Structurally distinct CTXs from the Pacific (P-CTX), Caribbean (C-CTX), and Indian Ocean (I-CTX) have been reported [92–100].

**Figure 1:**
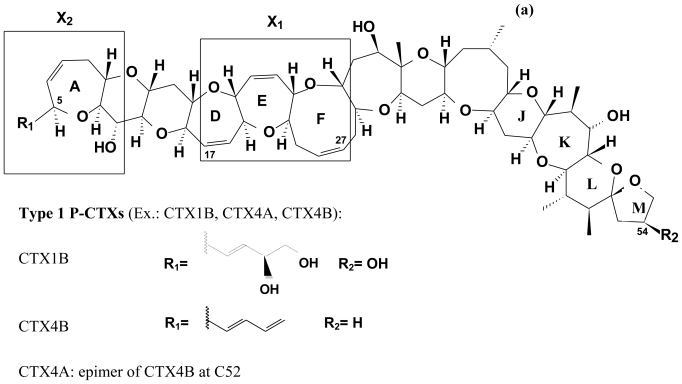

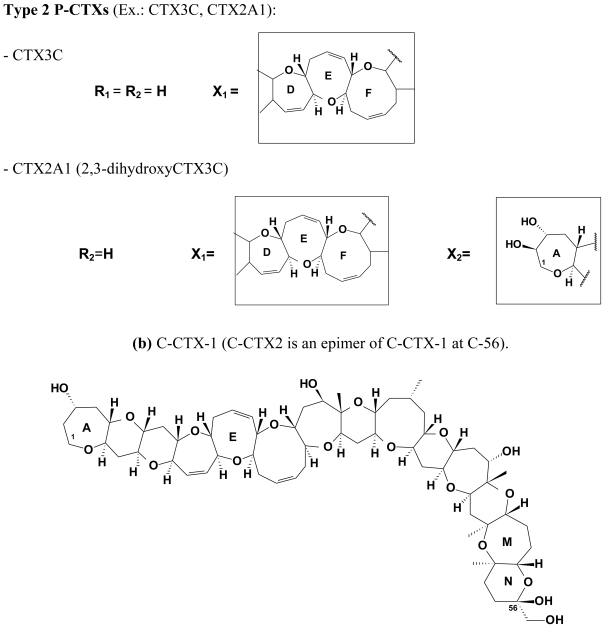
(**a**) General and specific structures of Pacific ciguatoxins (P-CTXs) (**b**) Structure of Caribbean ciguatoxins (C-CTX-1).

CTX-1B, a 60 carbon polycyclic ether, was the first CTX completely described in 1990 by Murata *et al.* [100]. Numerous congeners of CTXs, originating from fish and microalgae of the Pacific (P-CTXs), were further described. These congeners presented slight modifications of their lipophilicity. Occurrence of these different toxins in fish and microalgal samples vary. However, P-CTX-1 (P-CTX-1B) usually dominates toxin profiles in the carnivorous fish tissue of the Pacific [45,92]. In order to classify the different congeners of CTXs, Legrand *et al.* [101] proposed the distinction between two families of P-CTXs (types 1 and 2) according to the number of carbons and the structure of the E ring (See Table 1 and Figure 1). Two CTXs from the Carribean Sea (C-CTXs) were first isolated by Vernoux and Lewis [94], and further identified structurally in 1998 [94,102] (Table 1 and Figure 1). Additional congeners were later identified by Pottier *et al.* [97,98]. More recently, four Indian Ocean CTXs (I-CTXs) were isolated [96], but their structural determination remains to be established.

### 2.5. Toxicity and mechanisms of action

CTXs are extremely potent marine toxins with an LD50 in mice (i.p.) equivalent to 0.25, 2.3, and 0.9 μg kg^−1^ for P-CTX-1, P-CTX-2, and P-CTX-3, respectively, leading to the observation that the degree of oxidation is positively correlated with potency [45]. Recently, the 54-deoxyCTX was described as more potent than P-CTX-1 [22]. P-CTXs are more potent than the C-CTXs (LD_50_ of 3.6 and 1 μg kg^−1^ for C-CTX-1 and C-CTX-2) and I-CTXs (approximately 5 μg kg^−1^). No more than an estimated 1 ng P-CTX-1 per kilogram of body weight is needed to elicit the occurrence of mild CFP symptoms in humans [19].

CTXs directly target the voltage-sensitive Na^+^ channels, thus inducing many effects at the cellular and physiological levels, such as membrane excitability, release of neurotransmitter [103], axonal and Schwann oedema [104,105], increase of intracellular calcium [106], and blockage of voltage potassium channels [107]. CTXs stimulate Na^+^ entry [108] through specific binding to site 5 of the voltage-gated Na^+^ channel [109]. The affinity of the different congeners of CTXs for the voltage-dependent Na^+^ channel is proportional to the congeners’ respective intraperitoneal LD_50_’s in mice [110].

Extensive reviews on the mechanism of action of CFP exist [111]. We want to consider a recent hypothesis that explains possible chronic effects of CFP. The chronic long lasting effects of CFP actually seem to differentiate CFP from other marine toxin intoxications. Neurological symptoms are consistent with CTXs’ interactions with voltage-gated Na^+^ channels. However, all the symptoms produced during CFP may not be exclusively due to the blockage of the voltage-gated Na^+^ channels [112]. The chronic fatigue syndrome that may last for weeks or months [113] has been related to high nitric oxide (NO) production [114]. Penil and muscular relaxation, as well as L-type Ca^2+^ channel activation, were shown to be NO-mediated via the NOS pathway [115,116]. Up-regulation by CTXs of NO and inducible nitric oxide synthetase (iNOS) was recently confirmed by Kumar-Roiné [112]. As a matter of fact, P-CTX-1 was shown to induce a time- and dose-dependent increase in NO release within macrophage RAW 264.7 cells, through a high and prolonged increase in iNOS mRNA expression. It was further proposed that prolonged voltage-gated Na^+^ channel activity stimulates *N*-methyl-D-aspartate (NMDA receptor), resulting in Ca^2+^ influx, followed by constitutive NOS activation, thus leading to NO production. NO would in turn interact with the oxidative stress chain, resulting in iNOS gene stimulation, leading to further NO production. NMDA activation by CTXs is still not demonstrated, but brevetoxin-induced, prolongated voltage-gated Na^+^ channel activity has been shown to induce activation of the Ca^2+^-independent iNOS activity. The NOS pathway may provide insight into new therapies for CFP, such as NO scavenging or iNOS inhibition, although mannitol, as well as some traditional remedies, has been described as free radical scavengers [117,118].

## 3. Methodologies for CTXs Determination

### 3.1. Importance of CTX determination

CFP is the result of simultaneous exposure to a suite of closely-related but structurally distinct congeners (CTXs) with different intrinsic potencies and very low concentrations in fish. Indeed, it is well known that toxin profiles differ largely between herbivorous and carnivorous fish, and both at the species and individual levels.

For a number of marine toxins, data from human intoxication incidents and also toxicological data obtained in laboratory tests, (including biological (*in vivo* or *in vitro*) and physico-chemical assays using purified standards) have contributed to help regulatory authorities set a Maximum Permitted Level (MPL) for each of these toxins. Above this MPL, seafood must be banned from the market. For instance, based upon the evaluation of CTX content in fish samples using a mouse bioassay, an MPL of 0.01 ng g^−1^ P-CTX-1 equivalent toxicity was proposed for fishery products caught in the Pacific in 2000 [18,19]. This MPL was derived from estimation of CTX content in fish samples taken from meal remains after a mild outbreak of CFP. The value takes into account a 10x safety factor in order to address individual human risk and possible uncertainties in the assay accuracy [18,19] and in the assumptions of the amount of fish consumed. For Carribean fish, an MPL of 0.1 ng g^−1^ C-CTX-1 has been proposed [94]. In 2008, Dickey [119] presented an analysis of more than 100 outbreaks of CFP in the U.S. for the period of 1998–2008 in which exposure levels to CTXs were consistent with the MPL previously proposed for Pacific and Caribbean region fish [119,120]. These MPLs - established from analysis of fish tissue implicated in CFP - are currently used as acceptable levels to guarantee consumer protection [120].

One important issue regarding CFP management in a specific area consists of the discrimination of non-toxic from toxic fish specimens. Presently, this requires cost and the time-consuming procedures of extraction, purification, and determination of CTXs from the flesh of fish. Optimization of extraction and purification protocols to improve recovery of CTXs is still required. The development of methodologies that could detect CTXs with high specificity and sensitivity, and which are suitable for routine monitoring of food, is a matter of concern. Additionally, the confirmation of the presence of CTXs in food or in the blood of patients may help the clinical diagnosis of CFP [4], enabling differential diagnoses from other forms of ichtyosarchotoxism. Furthermore, purification of CTXs from the flesh of fish is of real interest for the identification of new CTX congeners and for the characterization of CTX profiles in different regions [24]. Finally, recovery of CTXs for reference production from fish material or for pharmacological studies in the elucidation of the CTXs’ mechanisms of action also requires the development of reliable methods of detection.

The abundance and distribution of *Gambierdiscus* spp., as well as CTX production by these microalgae in natural populations, are routinely monitored by some laboratories in ciguatera endemic areas. The occurrence of toxic (CTX producing) *Gambierdiscus* spp. blooms may be used as a bio-indicator of local ciguatera risk in a specific area [33,34]. Some time (months) after the occurrence of toxic *Gambierdiscus* spp. blooms, the appearance of CTX-containing herbivorous fish may occur [121]. Determination of CTXs in phytoplankton samples containing *Gambierdiscus* spp. may also have importance for geographical ciguatera risk assessment [70,122]. Determination of CTX production by *Gambierdiscus* spp. cultures, in parallel with taxonomic studies of the genus *Gambierdiscus*, is crucial for associating toxin production with a particular *Gambierdiscus* species [67,68]. Studying toxin production in cultures also helps to describe the kinetics of CTX production under specific, controlled laboratory conditions [77,122,123] or according to the physiological growth stage of *Gambierdiscus* spp. cells [69]. Production of gambiertoxins (ciguatoxin precursors e.g., CTX-4B) by *in vitro* culturing of *Gambierdiscus* spp. was proposed as an alternative supply of CTXs for reference material production [16].

In this chapter, the different strategies proposed for CTX quantification are considered. This includes describing sample preparation (for fish and microalgae) and the different existing methodologies for CTX quantification. Interestingly, and contrary to other microalgal toxins implicated in food poisoning, no recognized official method for the determination of CFP toxins exists.

### 3.2. Sample preparation for CTXs determination

An important issue when considering method development for marine toxins is sample preparation prior to toxin determination. Numerous protocols for sample preparation for research or monitoring purposes, or both, have been reported in the scientific literature for CTXs during the last decade. The nature of the sample, either of fish or microalgae, will have to be specifically addressed, with regard to the elimination of interferences and the expected toxin profiles. These two factors differ significantly between fish and microalgae. Methods for CTX determination that follow sample preparation may also determine the grade of purity of extracts required for the analysis. For example, purification of extracts through various Solid Phase Extraction (SPE) steps after solvent partition is often applied when analyzing CTX content using LC-MS/MS and CBA methods [119]. Another matter of concern is the time required, especially for routine monitoring purposes, for sample preparation to guarantee good CTX recovery. A rapid extraction protocol using LC-MS/MS analysis [124] has recently been developed to improve the usefulness of the screening of CTX-containing fish. This protocol is two–to-three times faster than the “long” procedures [125]. During another survey of ciguatera [33], an additional rapid sample preparation protocol was described for the screening of CTX-containing fish using the Receptor Binding Assay (RBA). We present here a selection of protocols published for the preparation of microalgal and fish samples containing CTXs. These methods are detailed below according to the detection method implemented following sample preparation.

#### 3.2.1. Fish samples

Fish sample preparation for CTX determination usually follows a general pattern derived from the first descriptions given by Scheuer’s [126] and Yasumoto’s teams [127] in the 1960s. Acetone or methanol based extraction is used for the recovery of lipophilic compounds from fish samples. Acetone extraction, being more efficient, is preferentially used, and is often implemented two or three consecutive times to optimize toxin recovery. A second step consists of the elimination of excessive fatty acids based upon solvent partition with hexane, diethyl ether, or chloroform (as in accordance with the protocol). Additional purification procedures using SPE have been proposed to improve the elimination of resulting fatty acids. Because fatty acids may vary in quantity and quality with the fish species, origin, or age of the fish, and because different CTXs may be present according to the geographical origin of samples, certain conditions of the purification steps, such as volume and solvent gradient, have to be modulated.

**Figure 2:**
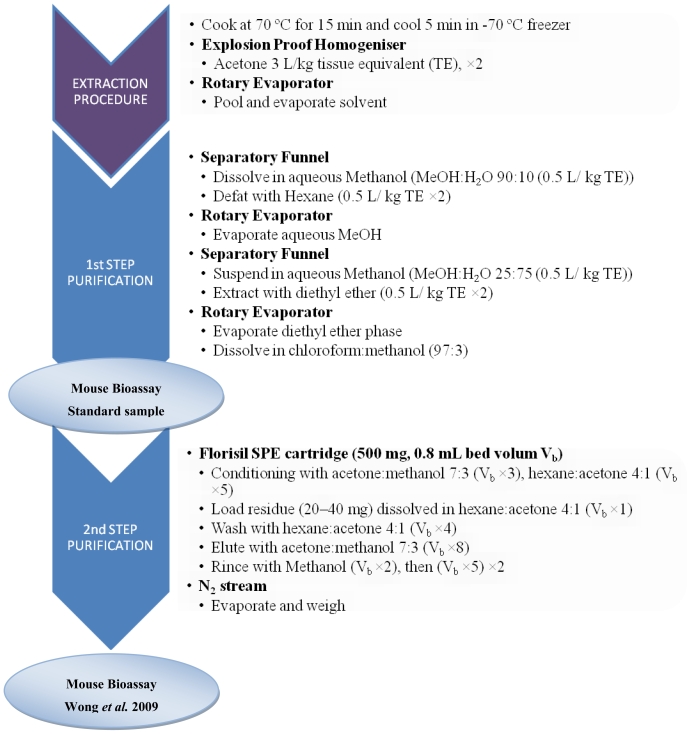
Solid-phase extraction clean-up of CTXs containing fish extract for use with the mouse bioassay according to standard preparation procedure ([128] and [131]).

The standard mouse bioassay (MBA) performed by intraperitoneal (i.p.) injection of the diethyl ether fraction obtained after liquid partition of fish extracts is often used for the surveillance and determination of CTX-contaminated fish [128–130]. Improvement of sample preparation to enhance detection and accurate determination of CTXs in the flesh of fish using the MBA was investigated by adding a clean-up step with Florisil SPE to increase the purity of the ether fractions [131] (Figure 2). According to these authors, this additional purification step enhanced recovery of P-CTX-1 to 76% *vs.* 63% with the standard purification protocol.

**Figure 3:**
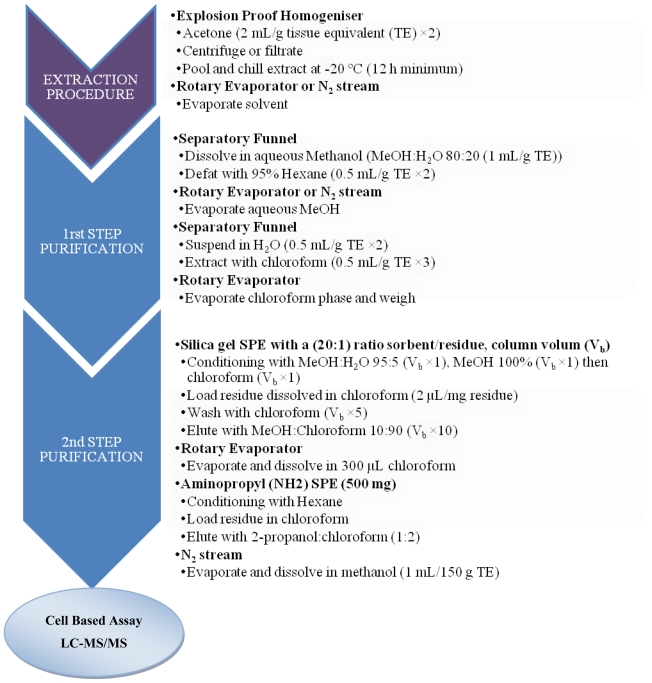
Extraction and purification of CTXs from fish samples (caught in the Pacific and Caribbean) for use with Cell Based Assay and LC-MS analysis [119].

Dickey *et al.* [119] proposed another purification protocol for CTXs to be used with CBA and LC-MS/MS analysis (Figure 3). This protocol is based upon the use of successive solvent partitions, followed by various SPE clean-up procedures. The method was reported to be successful for the clinical recognition and epidemiology of Pacific and Caribbean ciguatera [119].

**Figure 4:**
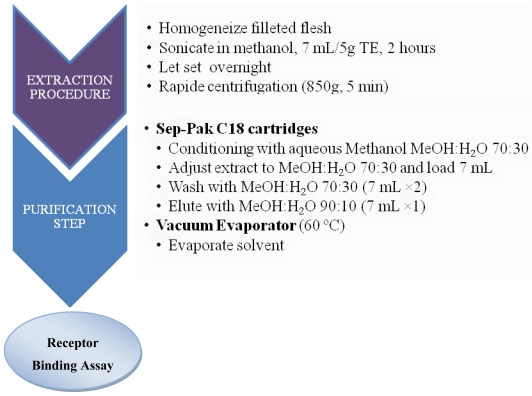
Extraction and rapid purification of CTXs from Pacific fish samples to be used with the Receptor Binding Assay [33].

Availability of flesh may be one limitation for the determination of CTXs in leftovers meals. In 2007, Darius *et al.* [33] described a rapid extraction procedure implemented for routine monitoring of ciguatera risk in French Polynesia that is suitable for RBA analysis. This procedure, based upon the extraction of 5 g of fish flesh, is applicable to both herbivorous and carnivorous fish of the Pacific (Figure 4) [33,34]. Extraction efficiency presented good concordance with standard extraction protocols for highly contaminated samples [132].

**Figure 5:**
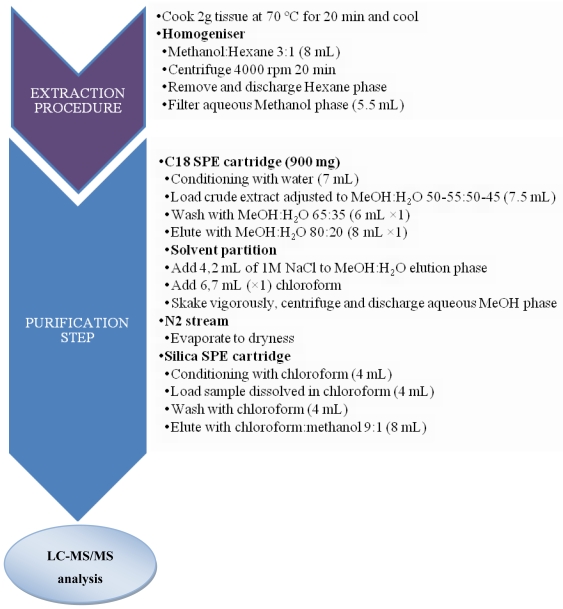
Rapid extraction and purification procedures for CTX determination in Pacific fish samples using LC-MS/MS analysis (CREM-LC-MS/MS) [124].

A CTX rapid extraction method (CREM) was developed by Lewis *et al.* [124] in order to reduce the time of sample preparation with the extraction of only 2 g fish flesh to be used with LC-MS analysis (Figure 5). The CREM was described as a successful procedure for P-CTX determination in Pacific carnivorous fish, allowing 95% recovery of spiked P-CTX-1 and 85% recovery of P-CTX-1 spiked at a concentration close to the limit of quantification of the method (0.1 ppb). According to these authors, CREM combined with LC-MS improves sensitivity and allows multiple analyses per day [124]. Stewart *et al.* [125] further adapted the CREM described by Lewis [124] with an additional methanol:hexane extraction step while omitting the final normal-phase SPE clean-up. This modified CREM not only reduced the time of preparation for fish extracts while requiring only a small quantity of fish flesh, but also showed an excellent reproducibility for P-CTX recovery from fish flesh, as well as a lower detection limit of 0.03 ng g^−1^ using LC-MS-MS [125].

#### 3.2.2. Microalgal samples

Sample preparation procedures for CTX determination in microalgae are still based upon the original extraction protocols previously described for CTX (or gambiertoxin) characterization from wild and cultured *G. toxicus* [92,100,133,134]. The first step consists of extracting cell pellets using methanol (aqueous or absolute) [33,68–70,84,122,134] or acetone [100]. According to the aim of the research and the grade of purity required, the resulting crude extracts may be further purified for the separation of CTXs from other concomitant toxins using liquid/liquid solvent partition with dichloromethane, hexane, or diethyl ether [33,68–70,133–135], separation through filtration of insoluble MTX in −20 ºC acetone [129], chromatography on SPE cartridges [33,69,70,100,133–135], or by high performance liquid chromatography (HPLC) [69,92,100,134].

One important aspect to consider while dealing with *Gambierdiscus* spp. extracts is the possible interference caused by MTX, another major toxic compound produced by *Gambierdiscus* spp. isolates, as underlined in previous studies [68,69,77,83]. It has been shown that MTX-derived toxicity may vary according to strain origin or culture conditions [69,83,123] and that considerable quantities of MTX (in toxicity equivalents) may occasionally take over the production of CTXs [83].

**Figure 6:**
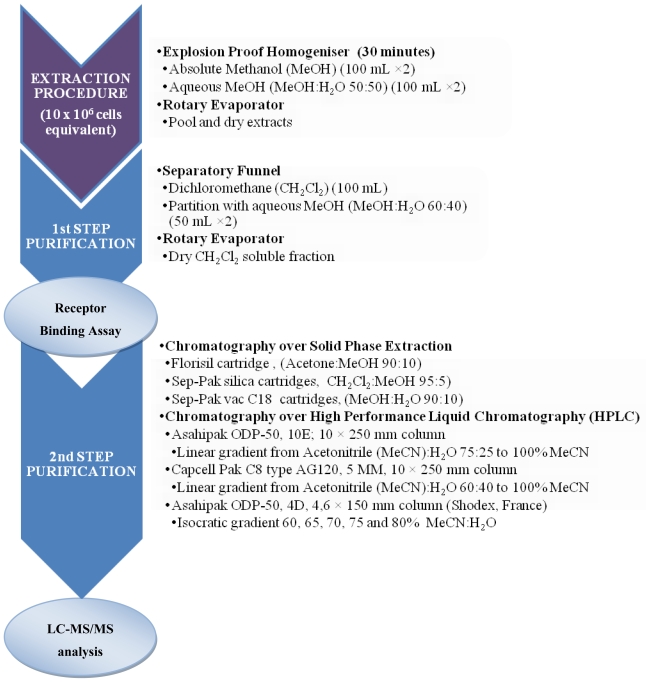
Extraction and purification procedure of CTXs from wild and cultured *Gambierdiscus* spp. cell pellets [33,68,69].

It is clear that the purification procedure for CTXs is highly conditioned by the presence of MTX, and may vary according to the type of sample. For instance, highly concentrated MTX samples may require the combination of solvent partition with chromatographic fractioning using SPE or HPLC so as to improve the separation of CTXs from MTX. Historically, MBA has been widely used for assessing the good separation of CTXs *versus* MTXs [69,83], although the presence of CTXs in the different fractions obtained may also be monitored using RBA [33] or CBA [136]. A recently developed CBA for the detection of MTX may help with the identification of the interferences MTX produce during CTX purification steps [137]. Figure 6 illustrates a protocol for CTX extraction and purification for use with the MBA and RBA [33,68,69]. In the case of further characterization of CTXs with LC-MS analysis, additional purification steps through SPE and HPLC fractioning are required [69]. This extraction procedure has been successfully used for the identification of toxic blooms of *Gambierdiscus* in field-monitoring programs [33], as well as for the assessment of the toxin profiles of highly potent *G. polynesiensis* strains [69].

### 3.3. Methods for CTXs determination

“Home-made” or traditional tests for discriminating safe fish from toxic ones should be briefly mentioned. These tests are widely used by local populations in ciguatera endemic areas, and include animal testing (e.g., feeding a piece of fish to a dog or a cat, observing avoidance of toxic fish by flies or ants, looking for the presence of a blood line at the tail of the fish [34], observing silver coins turning black on a hypothetical, cooked toxic fish, or feeling a sensation of tingling when rubbing the liver on the gums [34,138]). However, as stated by Banner *et al.* [138], these tests have very low credit and further discussion does not fit within the scope of this publication.

The high diversity and structural complexity of CTX congeners present at trace levels in different matrices (*i.e.,* microalgae, herbivorous and carnivorous fish tissue) have greatly hampered the development of reliable methods for their determination. In the course of developing CTX detection methods, one should consider the degree of specificity and sensitivity suitable for routine monitoring of CFP. An ideal universal method for CTX determination has yet to be developed, even though many methods, with varying success according to the application context, have been described.

Before addressing the descriptions of the different methods of CTX determination, it is important to remark that problems regarding the availability of CTX standards constitute an important limitation upon method development. Only a few laboratories produce purified CTXs [119] and commercial CTX standards do not exist. The limited availability of CTX standards and ciguateric reference material constitute major restrictions for the development, calibration, and validation of methods for CTX determination. These scarcities undermine the realization of pharmacological and toxicological studies that aim to elucidate the CTXs’ mechanisms of action and also limit the production of antibodies against CTXs.

CTX analogs can alternatively be obtained via the production of gambiertoxins (CTX precursors) by cultures of *Gambierdiscus* spp. [16]. However, the ability to produce CTX precursors seems to be genetically determined in *Gambierdiscus*, and limited to only certain strains of *Gambierdiscus* spp. [69,74]. For example, the mass culturing of a “super-producing” clone of *G. polynesiensis* allowed the purification of approximately 3.5 mg of pure algal CTXs from 2 × 10^9^ cultured cells [69], suggesting the need to increase efforts directed at the isolation of CTX “super-producing” clones for reference material production.

The synthesis of CTX to resolve the lack of availability of CTXs pure material appears as an ambitious challenge reflected by its large (3 nm long) and complex ladder-like structure [139]. In 2001, the total synthesis of CTX3C was achieved by Hirama *et al.* [139], based on highly convergent and efficient strategy for the assembly of structural fragments. This strategy was further improved by Inoue *et al.* [140,141] for a higher yield recovery. The 51-hydroxyCTX and CTX (CTX-1B) were also synthesized [142–144]. The total synthesis of gambierol, another toxin produced by *Gambierdiscus* spp. that is suspected to have a role in CFP [90,91], was also described [145]. Efforts directed at the synthesis of CTX congeners are necessary and may accelerate the preparation of anti-CTX antibodies.

Clearly, when selecting CTX determination methods, one should consider the frame of application, e.g., for food safety assessment, research, or diagnostic purposes. The development of simple and cost-effective individual tests (e.g., kits to be used by fishermen and consumers to check the safety of fishing products for commercialization or consumption, respectively), requires a different approach than what is needed by the CTX recognition methods used in research laboratories that look for accurate quantifications (e.g., for the diagnoses of cases of CFP). One can differentiate between methods that provide results on toxicity (e.g., a cytotoxicity response) from methods that provide the quantification of a specific compound (e.g., LC-MS/MS).

Comparative studies among the different existing methodologies have often presented good correlations. For instance, RBA values for CTX toxicity determination correlated well with those obtained using an analytical approach, MBA or CBA [4,146–148]. A comparative study between enzyme-linked immunoassay (ELISA) and CBA also indicated a high correlation between both methods [149,150]. The same remark applies to CTX determination using CBA in parallel with LC-MS/MS analysis [119,151]. All these methods show good results for the determination of CTXs at levels that can cause CFP in humans.

Some of the present strategies implemented for the screening and confirmation of CTX-containing fish may result from the combination of various complementary approaches. Dickey [119] and Dickey *et al.* [24,120] reported CBA to be a useful toxicological tool for the screening of toxic fish samples, to be then confirmed and the presence of CTXs quantified using LC-MS/MS analysis. The same approach was recently used during a survey of ciguatera along the Cameroon coastline [151].

The current methods for CTX determination are presented below. They include MBA, bioassays on animal tissue, *in vitro* neuroblastoma CBA (Neuro-2a CBA), pharmacological RBA, immunological assays, and physico-chemical analytical methods.

#### 3.3.1. Bioassays with animals: The MBA for CTXs

Attempts to use a wide variety of animals for the screening of ciguatoxicity in fish have been proposed but, excluding mice, bioassays using animals have met limited success [152]. *In vivo* bioassays consist of feeding or exposing animals to fish samples or direct injection of fish extract. Among these assays, mammalian based methods using mangoose [153] or cat [154] may produce symptoms resembling those of humans exposed to CTX-containing food. However, presently these methods are no longer used, as we have quite a good comprehension of CTXs’ mechanisms of toxicity. The implementation of such methods may be understandable in early toxicological studies or if new issues in toxicology need to be addressed. The MBA for CTX, further described, was first introduced by Banner *et al.* [153] and it is still the most widely used mammalian *in vivo* model for toxicity screening of CTXs. The chicken was also proposed as another model for a bioassay, showing higher sensitivity to CTXs than MBA [155] with easier handling. However, probably due to the lack of standardization, or the fact that a non-mammalian model may be not considered a good toxicological model, this model is not presently used, to the best of our knowledge. Invertebrate-based assays like the brine shrimp assay [151,156], mosquito assay [154], and diptera larvae assay [157] are low cost, simple detection methods that require low quantities of samples and can be used to screen numerous samples in a short time period. These assays are not widely used in laboratories responsible for the screening of ciguatera, probably because they lack specificity or because they may not be suitable for CTX quantification [24,158].

The mouse model is widely used in many diagnostic and research fields, such as drug development, cosmetic toxicological studies, and drugs, additives, or food contaminant testing [159]. MBA is still the current official detection method for most marine toxins, such as the paralytic and diarrheic shellfish poisoning toxins, which has been used for the protection of consumers for the past decades [159]. However, ethical pressure to avoid research with animals is supporting the development and validation of alternative or complementary methods (Directive 86/609/EEC) [160] for the elimination or decreased use of MBA’s in routine food safety assessments. With respect to ciguatera, MBA’s have been widely used for the screening of ciguatoxicity in fish samples. The MBA was first introduced by Banner *et al.* [153] and further revised by Yasumoto *et al.* [161] before its validation by Lewis and Sellin [162] as a reliable method for the selective determination of CTXs. The method proposed by Lewis [129,130] allows the quantification of natural levels of CTXs in the fish flesh to up to 20 mg of ether extract. The relationship between the dose and time to death is used to quantify toxicity of the extract. Usually the mouse unit (MU) is used and it is defined as the lethal dose (LD) of a toxin that kills a 20 g mouse in 24 h after i.p. injection. One MU is equivalent to 5 ng, 18 ng and 48 ng for Pacific CTXs, P-CTX-1, P-CTX-2 and P-CTX-3, respectively [45,162] and 72 ng for pure Caribbean CTX-1 [147]. Dose and time to death relationship for a mix of ciguatoxins typically found in carnivorous fish is defined according to the equation: log MU = 2.3log(1 + 1/T), where T is the time to death in hours [46]. It was formerly suggested that any fish containing above 2.5 MU/100g should be avoided as food [161] since ciguatoxins are potent neurotoxins that may have long-term neurological effects.

Protocols for the MBA are well described in Lewis [129,130]. Generally, mice of 20 ± 2 g of either sex are marked for easier identification and each extract tested is assayed in duplicate. An aliquot of the extract is evaporated until dry, weighed, and re-suspended in a physiological solution (0.8–0.9% NaCl, 1–5% Tween 60) at a final volume of 0.5 mL for each mouse to be injected. Injection of extracts occurs in the ventral peritoneum of mice (i.p injection) using a fine short needle syringe. Mouse handling during injection is detailed in Lewis [129]. At this stage, the suggested amount of fish extract injected per mouse may vary according to the author. Dickey *et al.* [163] recommend an injection volume of 0.5 mL of physiological solution containing fish extract with a residue weight that should not exceed 20 mg. The dose of extract injected per mouse could, for example, correspond to muscle tissue fresh weight equivalents of 45, 90 or 180 g. Control mice received Tween/Saline solution only. For each marked mouse, in addition to the weigh, sex, and quantity of extract injected, it is necessary to control the time of injection, signs of intoxication, rectal body temperature (optional), and time to death [129]. Mice should be observed intermittently for up to 48 h, and should be studied more closely during the first two hours after injection [129]. Sub-lethal doses of CTXs should be associated to a growth depression within four days of injection [130,131]. Signs of ciguatoxicity include: inactivity, piloerection, vasodilatation in ears, cyanosis of tail, feet, and muzzle, unsteady gait, tremor/convulsive jumping, and death. However, reliable characterization of CTXs requires observation of hypothermia below 33 ºC, dyspnea with gasping, severe diarrhea, hypersalivation, and lacrimation [162]. If short survival lengths are observed, doses should be reduced to observe symptoms typical of CTXs. Sensitivity of mice to CTXs may vary according to the strain and housing conditions: it is suggested to establish a specific dose time to death relationship using mice from the breeding colony and for each source of CTXs to be assayed [130]. However, the limited amounts of pure CTXs make it difficult to establish the dose time to death relationship.

The MBA is probably the most reliable *in vivo* bioassay system; it has many references of its application in research, including those that support its ability to clinically recognize ciguatera, and to a lesser extent, to monitor market fish. Presently, this *in vivo* toxicological assay is still currently being used for screening of CTX bioactivity in fish and microalgal extracts [69,128,131,147]. As an example, the MBA was implemented for surveillance of CTX-contaminated fishes in the market and of import sources in Hong-Kong so as to identify the problematic fish species and the ciguatera affected areas of importation [128]. Objective parameters for the MBA were adopted for CTX determination, especially with respect to the detection of sub-lethal amounts of CTXs, with the measure of body weight depression at day 4 after injection. Sublethal doses were estimated to range between 0.18 and 0.45 MU 20 mg^−1^ ether extract [128]. According to Wong *et al.* [131] (Figure 2), a modification in the purification procedure with respect to standard protocols allowed for the elimination of unnecessary lipid matrix. This modified procedure was reported to induce a more rapid death of mice compared to mice injected with non purified diethyl-ether fractions (in accordance with the standard purification protocol) [131]. This result suggested that the matrix inteference suppresses or retards the toxic effect of CTXs through a possible interaction of matrix compounds with CTXs at their target sites [131]. Such a modification in the purification procedure for use with the MBA may improve the detection of CTXs from suspected cases and borderline case samples. In the case of microalgal extracts, the MBA with a dichloromethane fraction of cultured *G. polynesiensis* allowed for the detection of CTX bioactivity at different growth stages of the culture, thus allowing for the detection of a peak toxicity value at the onset of the senescent phase. This peak value was estimated at 43 × 10^−5^ MU cell^−1^ [69]. MBA also helped for the purification of pure algal CTXs directly produced by *G. polynesiensis* through the location and quantification of CTXs bioactivity in each fraction resulting from the different purification steps [69].

The MBA allows a reasonable and sensitive detection of CTXs [164] whenever signs of intoxication of mice are consistent with CTXs [19], but its utility is limited by the requirement of a dose-response curve [130,164] with purified CTXs for accurate quantification (since the dose-response curve is not linear) [130]. Additionally, free unsaturated fatty acids (e.g., Docosahexaenoic acid, DHA) interfere with the MBA. Despite its extended application, its need for the maintenance of animal colonies, its cumbersome execution, and its ethical concerns, has favored its replacement with alternative or complementary methods that are more suitable for laboratories in charge of ciguatera management. As an example, the MBA has been used since 1990 by the FDA Gulf Coast Seafood Laboratory for assessment of suspected toxic fish samples to support clinical diagnostics until its replacement in 1994 by the *in vitro* bioassay (described below) [119]. The MBA is unsuitable as a market test for individual checking of CTXs; however, it remains a toxicological tool that is accessible to many laboratories and is capable of effective clinical recognition. This is particularly important for those who lack access to other validated alternative methods.

#### 3.3.2. Bioassay with tissues

The contractile activity of CTXs was investigated on atria isolated from guinea pigs and was described to induce a prolonged, positive ionotropic effect upon the electrically driven atria [165]. This effect was further refined to allow the detection of CTX containing fish extract based upon the guinea pig atrium assay. The assay consisted of measuring the ratio between the amplitude of contraction of the guinea pig atrium after the addition of extract with the initial amplitude of untreated atrium [166]. This assay proved useful when CTXs were present at high concentrations, and has been used for the confirmation of ionotropic activity in fish extract [150]. However, the assay is likely to be suitable only for research purposes; the availability of tissue produces a technical limitation.

#### 3.3.3. *In vitro* bioassays: CBA

*In vitro* cell models provide important tools for studying the effect of toxins in cells isolated from different tissues and for comprehending the mechanisms of action in cells that present specific targets (e.g., receptors) for the toxin in question. *In vitro* approaches allow more comprehensive toxicological profiles, which are of additional value beyond the single “hazard identification” [167]. Cells respond rapidly to toxic stress through the alteration of metabolic rates, cell growth, or the transcription of genes that control basic functions. Consequently, the CBA has been developed for the analysis of food designated for human consumption [167]. The development of cytotoxicity assays for toxin detection has important methodological implications and represents a clear advancement in hazard identification because the use of cultured cells is likely to replace the use of living animals [168]. Cytotoxicity can be defined as the potential of a compound to induce cell death [167]. Numerous endpoints have been developed to assess cytotoxic effects. The [3-(4,5-dimethylthiazol-2-yl)-2,5-diphenyltetrazolium] MTT test [169] is the most widely used endpoint for cytotoxicity screening of CTX-containing fish extracts [119,150,151] and of microalgal extracts [70,136]. MTT is reduced by the mitochondrias of metabolically active cells into a blue formazan product that can be easily quantified using a standard spectrophotometer [169]. Such a colorimetric assay requires minimal processing and allows for accurate and reproducible quantification of toxic effects [170].

The specificity of the CBA for one particular group of toxins is a challenge for its application in toxicological contexts [168]. Having a specific assay for a targeted family of toxins is crucial for diagnostic purposes because several toxic compounds can be present within natural samples, or the sample itself may cause cell death. The lack of specificity in cytotoxicity assays may hinder their application in screening marine toxins in natural samples. However, in another aspect, a cell-specific toxicity has been recognized based upon sensitivity of the cell model or impairment of specific functions [167]. Specificity of the CBA may also be achieved with modifications introduced regarding the action of toxic compounds that enhance or hinder the compounds’ cytotoxic effects. For example, specific agonists or antagonists of some toxins have been used as described for the detection of several neurotoxins, such as saxitoxin, brevetoxin, ciguatoxin, and palytoxin [148,171–175].

**Figure 7:**
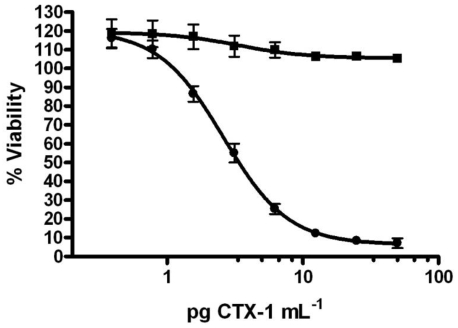
Neuroblastoma (Neuro-2a) cells exposed for 24 hours to P-CTX-1 in the presence or absence (▪) of ouabain (0.1 mM) and veratridine (0.01 mM). The proportion of O and V produced an approximate 20% cell mortality. No toxic effects of P-CTX-1 were measured when Neuro-2a cells were not treated with O and V (▪). P-CTX-1 is toxic to Neuro-2a cells in the presence of O and V (●) with 50% of cell mortality produced with a concentration of 3.3 pg CTX-1 (IC_50_ = 3.3 pg P-CTX-1 mL^−1^) [195].

As for the application of CBA to marine toxins, the Neuro-2a neuroblastoma cell model (Neuro-2a) was first proposed to evaluate numerous neurotoxins, such as brevetoxins, ciguatoxins, tetrodotoxins or saxitoxins, which target voltage-gated sodium channels (VGSC) [176]. Binding of neurotoxins to their receptors on the VGSC modulates the conformation of these proteins, which cause an activation or blockage of the sodium influx [176]. However, the diversity of ion channel systems in different cell types may compensate for these changes such that the action of neurotoxins alone is not sufficient to cause cell death in many cell types. To address this issue, it was proposed that the addition of other toxic compounds active in sodium influx can result in disregulations of the intracellular Na^+^ concentration [177]. For example, ouabain (O) blocks sodium efflux through an inhibition of the ATP dependent Na^+^/K^+^ pump [178] and veratridine (V) increases Na^+^ permeability through a blockage of the voltage-gated Na^+^ channel in the open position [176]. The combined action of both O and V results in an increase of intracellular Na^+^ concentration that may result in cell death, depending upon the concentration tested. The addition of VGSC toxins that activate sodium influx (e.g., ciguatoxins and brevetoxins) increases the toxic effects induced by O and V, leading to higher toxic effects with respect to the control. The effect of the addition of VGSC toxins that block sodium influx (e.g., saxitoxins or tetrodotoxins) counteracts the toxic effects of O and V, resulting in less toxic effects with respect to the control. Comparison of the cell response after exposure to natural samples in presence or absence of O and V allows for the detection of neurotoxins and for their characterization in accordance with the type of cell response obtained (*i.e.,* the increase or decrease in toxic effects elicited by the treatment with O and V). The use of sodium channel blocking toxins (O and V) led to the development of various CBAs for the detection of neurotoxins [171,172,175,179–181]. The O/V dependent Neuro-2a CBAs, which are specific for neurotoxins with the MTT test as an endpoint for cytotoxic effect quantification [172], are probably the best established CBAs for the detection of neurotoxins in natural samples [171,172,175,179,180,182–184] and are actually the most widely used [119,136,148,150,151,173,174,184] **(**Figure 7). A Neuro-2a CBA ready-to-ship kit, consisting of a modification of the Neuro-2a CBA with shippable plates and reagents to be used by unspecialized laboratories, was developed for the determination of VGSC blocking toxins in 1999 [185]. But it proved to be unsuccessful, probably due to a lack of stability during shipment or unreliable laboratory conditions.

Other cell lines, including primary cell cultures, have been used to identify CTX mechanisms of action or to confirm and quantify the presence of CTX. For example, one can study these cells with the patch-clamp method [186,187] or a confocal laser scanning microscope to identify structural changes [104]. Some cell strains, such as the human embryonic HEK cell line or the neuroblastoma x glioma hybrid NG108-15 cell line, have been used for studying CTX’s modes of action or for studying their suitability for CTX determination by using the same approach as the one used with Neuro-2a cells (O/V dependent CBA) [106,188–191]. However, the current tendency of laboratories working on CTX determination using the CBA is to focus upon the Neuro-2a cell line. This is mainly due to the easy handling of this cell line, its elevated sensitivity to CTXs, and the accuracy in routine screening of CTXs compounds [119,120,136,148,150,151,173,192–194] (See Figure 7). Although the use of other cell models is not to be discarded, the use of the Neuro-2a cell line as a reference model is likely to be used in inter-laboratory comparison exercises for validation purposes and standardization.

Recent studies have described in detail protocols for the O/V dependent Neuro-2a CBA for CTX determination following either the original description given by Manger *et al.* [172] or with some modifications [119,150,174]. Briefly, Neuro-2a cells are maintained in 10% Fetal Bovine Serum (FBS) RPMI medium at 37 ºC in a 5% CO_2_ humid atmosphere. For experiments, cells are plated in 96-well microplate in 5% FBS RPMI medium at a density ranging from 20,000 [151] to 100,000 cells per well [119] according to the author. After a 24 h incubation, one half of the plate receives treatment with O and V at a 10:1 proportion; concentrations of O and V may vary according to the author [119,148]. It is recommended to treat cells first with O and then with V, since the low pH of the solution of V may inactivate O. The natural sample or CTX standard to be tested is usually first evaporated to dryness to completely remove the solvent, and then dissolved in 5% RPMI medium. The resulting solution is serially diluted in RPMI medium and an aliquot of each dilution is directly added into the well of both halves of the plate (with and without O and V treatment) in order to compare cell response in the presence and absence of O and V treatment. An equal volume in each well is reached using phosphate buffer solution. When checking for the presence of CTXs in fish samples, it is important to establish the maximum concentration of tissue equivalent (TE) to expose, since excessive matrix may be toxic to the cells. The quantity of matrix that one can expose varies according to the sample preparation procedure. For example, Dickey [119] used 2 g TE mL^−1^ and Dechraoui [148] recommends no more than 20 mg TE mL^−1^. For a quantitative estimation of neurotoxins present in natural samples, calibration of the assay with CTX standard(s) is necessary and recommended for each day of the experiment, since cell sensitivity may vary according to the day of experiment. After a 20 to 24 hour incubation of cells with CTX standard(s) or sample, cytotoxic effects are evaluated using the MTT test. Absorbance values are expressed in percentage of effect or viability with respect to controls (with and without O and V treatment). The quantitative estimation of equivalents of CTX in samples may be obtained using the substitution of the concentration of standard material that induced 50% of effects for the concentration of sample that induced 50% of effects [119]. The quantification of CTX compounds within samples presenting non-specific toxicity (toxic effects observed in the absence of O/V treatment) is achieved through a subtraction of both equivalents of CTX at the 50% of effects with and without O/V treatment [77]. The subtraction of unspecific toxicity for CTX quantification in microalgal samples may be used when interferences due to the presence of MTXs are suspected.

The Neuro-2a CBA has been widely used for discriminating between toxic and non-toxic fish samples [119,148], mainly for clinical recognition and for determining the epidemiology of ciguatera. It has proven to be a robust, reproducible, and high throughput method for studying CFP cases [119]. Its high sensitivity has allowed for the detection of small quantities of CTX. This is important as the IC_50_ for Caribbean CTX-1 is as low as 0.29 pg mL^−1^ [119]; additionally, the quantification in fish tissue can be as low as 0.039 ng CTX-1 g^−1^ [148]. The application of the assay has also been extended to the detection of CTXs of algal origin [70,136]. A chromatographic fractioning of natural samples (fish and microalgae) through SPE or HPLC, combined with the O/V dependent Neuro-2a CBA, may evidence CTX activity consistent with its retention time [119,148], and help with the separation of concomitant toxins [119,136]. The high screening capacity of the method almost finds application in the CFP monitoring program. As an example, 64 fish from the Cameroon coastline were screened using the O/V dependent Neuro-2a CBA, allowing for the detection of three toxic fish. This detection was further confirmed for CTX via analytical analysis. This result showed for the first time the health hazards associated with ciguatera along the southeastern Atlantic coast of Cameroon. However, as in any toxicological test, this assay will not discriminate amongst different neurotoxins acting in the same manner. Thus, confirmation of the identity of the toxic compounds using analytical approaches (further described) is required, whenever possible. This assay is part of the two-tiered protocol proposed by Dickey for the reliable determination of CTX in the routine screening of CTX-like activity in Caribbean and Pacific fish samples associated to human intoxication.

*In vitro* bioassays have been proposed as an alternative or complementary approach to animal testing in regulatory toxicology most notably in the field of chemicals, where they now succcessfully replace acute toxicity tests, such as the *in vitro* phototoxicity test or skin corrosivity tests [196]. In this respect, the Neuro-2a CBA shows great potential as a likely candidate in the quest for a validated reference method of CTX assessment in fish samples.

#### 3.3.4. Immunoassays

By virtue of their theoretical advantages high specificity combined with the high-resolution signal amplification of the antigen-antibody (Ag-Ab) reaction Ab-based assays have been considered as a very promising approach for designing a rapid, reliable, and cost-effective method for mass screening of fish prior to consumption. However, production of specific Abs to CTXs has been greatly hampered by CTXs’ extreme scarcity, toxicity, and chemical complexity [99,100,102,134,197]. Historically, Hokama's group in Hawaii [198] was the first to report the production of sheep polyclonal Abs (PAbs) to a partially purified CTX (CTX-1B). Some years later, Hokama and his co-workers were able to obtain mouse monoclonal antibodies (MAbs) to okadaic acid (OA) and CTXs [199,200] that could be produced in a potentially limitless and homogeneous supply. PAbs were first used in a tentatively improved radioimmunoassay (RIA) [166,201] and in the enzyme-immunoassay (EIA) [200,202] format assays. Subsequently, MAb-based assays were claimed to yield higher sensitivity and specificity [199,200,203,204]. Later on, numerous trials have been undertaken to validate such tests with field or clinically implicated fish samples [203–206], but these results have raised some controversy [163]. The CTX detection kit, «CIGUATECT™», which had been commercially available, was evaluated against the MBA for CTX determination with inconclusive results [163]. Presently, a new kit for CTX determination, the “Cigua-Check” is commercialized, although no information on its validation procedure is available.

Following these pioneering studies, in the more recent past, a great deal of work has been done in this field. This has been facilitated through the use of synthetic ring fragments of CTXs to overcome the scarcity of the natural toxins and via the advent of modern molecular biology techniques, which can provide a set of immunological reagents whose specificity and affinity can be improved.

Up to now, in spite of many attempts, there is no fully validated immunoassay screening that is commercially available for ciguateric fish, but promising results have been obtained by Hirama’s group in Japan [207–214]. As a general rule, in the course of producing Abs specific to Ags in laboratory animals, several critical steps must be carefully evaluated and planned such as: (i) the preparation and analysis of the Ags; (ii) the selection of the animal species in order to raise either only PAbs (e.g., sheep or rabbit) or PAbs plus MAbs (e.g., Balb/c mice); (iii) the selection of the adjuvant (e.g., Freund’s adjuvant); (iv) the development of an immunization schedule (*i.e.,* determine the dose, volume, route, number and site of injection, and intervals between injections); and (v) the monitoring of the Ab response (e.g., ELISA format assays). In this review, only some of these aspects will be presented.

From an immunological standpoint, CTXs are considered to be haptens, *i.e.*, low molecular weight compounds, which contain one or more antigenic determinants (epitopes), but which are unable to trigger an immune response *per se*, unless covalently conjugated to a large molecule acting as an immunogenic carrier. In a way that is similar to the haptens, there is a large diversity in the physico-chemical nature of the carriers, and useful ones include polymeric macromolecules, which can be natural (e.g., polysaccharides or proteins from bacteria or viruses) and semi-synthetic or synthetic materials (e.g., polyamino acids or resin beads) containing one or more functional groups to which a reactant moiety can be attached. For a comprehensive review on Ab production methods, the reader can refer to Harlow and Lane [215].

Although a large number of chemical or photochemical coupling procedures were already available at the time of the first immunochemical studies on CTXs, their adaptation or the design of new ones have remained crucial for many years, to develop simple, reproducible, and low-cost methods applicable to poorly available lipophilic haptens, such as CTXs. In fact, considering the numerous studies of Hokama and his co-workers [199,200,203–206], it must be remembered that the carbodiimide coupling procedure employed to obtain CTX-protein conjugates is not fully adequate in the absence of a carboxyl group. In 1989, the elucidation of the structure of the main congener P-CTX-1B revealed that the only functional group available was a primary hydroxyl located on a 4-C side chain of ring A [216]. In this context, our initial immunochemical knowledge of CTXs mainly came from experiments performed with more available hydroxylated polyether compounds, such as PbTxs [217–221], monensin [222], or even cholesterol [223]. Such experiments required a three-step procedure: (i) the formation of a hemisuccinate derivative of these hydroxylated haptens; (ii) the activation of the newly formed carboxyl group; and (iii) the conjugation to the protein.

As it has been consistently reported, a critical epitope density (*i.e.*, hapten/carrier molar ratio) of the conjugate is necessary to trigger a strong specific immune response in immunized animals [215], therefore conjugates must be carefully analyzed by various methods. These methods first require the separation of the conjugate from the uncoupled haptens by basic techniques (e.g., extensive dialysis against the buffer, filtration through Sephadex G25, and salt or solvent precipitation). After this, conjugates are characterized by conventional methods e.g., UV analysis, radioactive incorporation of labeled hapten, di- or trinitrophenylation, and gel electrophoresis, or more sophisticated procedures, such as gas chromatography, high performance capillary electrophoresis, matrix-assisted laser desorption ionization time-of-flight mass spectrometry (MALDI-TOFMS), and high-performance liquid chromatography (HPLC). However, most of these techniques are time-consuming or exceedingly complex. Therefore, a rapid, cheap, and simple technique based upon a modification of the Habeeb’s trinitrophenylation method [224] was designed for poorly available haptens, such as CTXs [225].

In the process of immunization of laboratory animals with a hapten carrier conjugate, haptens act essentially as epitopes for binding to B cell surface receptors (membrane-bound immunoglobulins); this is a necessary step for B cell activation. In addition, the carrier provides not only distinct B cell-specific epitopes, but also class II T cell receptor binding sites, which are required to trigger T cell helper function, which in turn induces the differentiation of B cells into Ab-producing cells.

Finally, it should be stressed that only a small fraction of the Abs produced in response to such multiple antigenic stimulation and complex cellular interactions are capable of binding to free haptens. For this reason, to evaluate the quantitative and qualitative Ab response, a soluble conjugate of the hapten with a heterologous carrier protein must be used in different immunoassay formats. The general procedure requires a succession of washing steps and incubation periods with immunochemical reagents. Firstly, using a direct sandwich ELISA procedure, Ab-containing fluids can be titrated using a 96-well ELISA plate coated with a fixed concentration of the hapten-heterologous conjugate. Bound Abs are revealed by the successive addition of the secondary Ab conjugated to an enzyme (e.g., horseradish peroxidase or HRP) and its substrate (e.g., *o*-phenylenediamine or OPD). Finally the signal is recorded on a microplate reader designed for colorimetric or fluorimetric assays, depending upon the physico-chemical nature of the product generated by the enzyme-catalyzed reaction. Secondly, to confirm the binding of the Ab to the free hapten, competitive inhibition ELISA (CIEA) is performed: a fixed and limiting amount of Ab is pre-incubated with increasing concentrations of the free targeted hapten. Afterwards, the mixture is dispensed into 96-well ELISA microplates coated with the hapten-heterologous carrier conjugate. Bound Abs are also revealed as previously described. This simple CIEA format enables the determination of parameters, such as the dissociation constant (K_D_) [226] and the limit of detection (LOD), which is calculated by assuming that a decrease of three standard deviations from the mean signal without inhibitor is significant (n ≥ 12).

Extensive analyses of toxin profiles supported by LC-MS have revealed that different fish species always contain a mixture of very low levels of CTX congeners. These congeners mainly differ by the number and position of substituents located on the terminal A and M rings, and by the size of the E ring [102,197,227] (See Figure 1).

Therefore, as a good alternative to the lack of pure CTX, conjugates of the JKLM ring fragment [228], a carboxylic derivative of the right-hand tetracyclic portion of CTX-1B, were prepared with bovine serum albumin (BSA) and ovalbumin (OVA) for immunization and the monitoring of antibody responses, respectively [229]. In this study, conjugates were prepared according to a bulk procedure (3–5 mg of hapten) via an activated ester method in a semi-organic medium or according to a microscale technique (300 μg of hapten) via the formation of a mixed anhydride in a reversed micellar medium. Both coupling methods yielded epitope densities that were within the same range: 20 and 12 for BSA and OVA conjugates, respectively. Monitoring of mouse PAb response proved that a long-term immunization schedule (injections spaced at four-week intervals) was more efficient than a short-term protocol (injections spaced two weeks apart). Indeed, when using a CIEA format assay, the long-term related PAbs exhibited high affinity (K_D_ = 7 × 10^−9^ M), but narrow specificity, whereas the short-term associated PAbs showed less affinity (10-fold factor), but greater immunoreactivity to JKLM-related natural compounds (*i.e.*, CTX congeners). No cross reactivity to PbTx-3, OA, monensin, or other polyether compounds was detected. With the aim of increasing the sensitivity of this assay, a biotin-avidin amplification system was used, which decreased the LOD for JKLM to 1.23 × 10^−9^ M.

Following this first demonstration of the relevance of using synthetic ring fragments of CTX-1B instead of the natural congeners, Hirama’s group [207–214] pursued their synthetic work to prepare suitable immunogenic conjugates of carboxylic derivatives of various CTX ring fragments via the bulk activated ester method. Analysis of the conjugates was performed by MALDI-TOFMS and the epitope density of the conjugates was always found to be compatible with good PAb response and production of MAbs in Balb/c mice. By using two ABC-ring fragments of CTX-1B coupled to BSA [208], they developed an elegant and rapid MAb screening system based upon surface plasmon resonance spectroscopy. This study stressed the importance of the A-ring side chain of CTX-1B for triggering adequate the mouse immune response. However, this conjugate was found to be poorly immunogenic (K_D_ = 6 × 10^−7^ M). A milestone on the way to the development of a valuable immunochemical assay for CTXs arose from a double synthesis-based approach, which allows the setup of a direct sandwich ELISA for CTX-3C [210]. Pentacyclic ring fragments of the left and right wing of this CTX congener (namely ABCDE and IJKLM, respectively) were coupled to the keyhole limpet hemocyanin (KLH) and BSA. Following mouse immunization with the two KLH conjugates, several MAbs were produced. Two were selected on the basis of their strong reactivity towards their corresponding ring fragment and also to CTX-3C, and also because of the absence of cross-reactivity to the non-corresponding ring fragment and to other structurally related marine biotoxins, including OA, PbTxs congeners and MTX. Therefore, a direct sandwich ELISA based upon a capture MAb 10C9 (K_D_ = 2.8 × 10^−9^ M for ABCDE) and a detector MAb 3D11 (K_D_ = 1.22 × 10^−7^ M for IJKLM) conjugated to HRP was proposed with a LOD of 5 nM for CTX-3C. The authors concluded that this strategy was expected to be generally applicable to all CTX congeners. This assumption was confirmed three years later by the generation of MAbs against the common right wing of CTX-1B and 51-hydroxyCTX-3C by using a KLH conjugate of the synthetic HIJKLM ring fragment [213]. MAb 8H4, which strongly binds to 51-hydroxyCTX-3C (K_D_ = 7.5 × 10^−8^ M for the right wing fragment) was found to discriminate between compounds that did or did not contain the 51-OH group. Similarly, a direct sandwich ELISA was proposed based upon the capture MAb 10C9 (K_D_ = 2.8 × 10^−9^ M, for the shared left wing fragment of CTX-3C and 51-hydroxyCTX-3C) and a new detector MAb 8H4 (K_D_ = 7.5 × 10^−8^ M for the right wing of 51-hydroxyCTX-3C) conjugated to HRP. A LOD of only 1 nM for 51-hydroxyCTX-3C was obtained. Finally, more recently, Tsumuraya and his co-workers [212] again used MAb 10C9 (directed to the left wing of CTX-3C) and another MAb 3D11 as a detector (K_D_ = 1.22 × 10^−7^ M for the right wing of CTX-3C), and obtained a LOD of 5 nM for CTX-3C.

Another study of interest performed by Hirama’s group described the production of recombinant Ab fragments (rFabs) to the ABCD ring fragment [209]. Starting from the deduction that the epitope recognized by MAbs in the CTX molecule encompasses more than three rings, mouse MAbs raised to ABC-KLH conjugates were genetically modified using phage-displayed Abs technology. In a CIEA study, three carefully selected rFabs displayed K_D_ values for free ABCD in the range of 2.4 × 10^−5^ M to 5.0 × 10^−5^ M. Moreover, rFab 1C49, exhibiting the highest affinity for ABCD, was also found to react to CTX-3C, though to a lesser extent. These last experiments and other studies [211,214] confirmed the importance of using more than four-ring fragments as synthetic haptens in designing valuable hapten-carrier conjugates for the production of high affinity and specific MAbs to CTX congeners.

Finally, it is worth mentioning the continuing efforts of Hokama’s group to improve their previously described format assays using an anti-CTX MAb [199,200,203,204]. Keeping in mind that their membrane-based assay successfully allowed crude polyether toxin binding following a methanol extraction from fish flesh, but was unable to detect pure CTX in methanol, they adapted their bamboo stick-based assay to allow better binding to both types of toxic fractions [230]. This assay enabled the detection of P-CTXs and C-CTXs in the very low range of 0.078–5.00 ng/mL (*i.e.*, 7.8 × 10^−11^ to 5 × 10^−9^ M, assuming an average MW of 1000 Da for CTX congeners) with no cross-reactivity to OA, palytoxin and domoic acid. In another attempt, Hokama and his co-workers developed a sandwich ELISA for detection of CTX [231] using a capture anti-ABCD CTX ring fragment raised in chicken and a mouse MAb directed against JKLM ring fragment conjugated to HRP. A CIEA format enabled a LOD of approximately 5 pg mL^−1^ of equivalent CTX per mg of extract (corresponding to 5 × 10^−12^ M), which lies in the range of the neuroblatoma cell assay. Moreover, the CIEA yielded consistent results when compared to this assay using flesh extract of fish caught *in natura* or raised in aquaculture farm [149,150].

The simplicity and low-cost of immunoassay, coupled with its speed and/or high through-put sample processing, make it an effective method for monitoring the toxicity of fish prior to consumption. Nevertheless, it should be remembered that its applicability should not only rely on the affinity and specificity of the Abs for their target molecules, but also on the availability of simple and rapid sample clean-up and concentration procedures.

At a single laboratory level, a fully acceptable CTX immunoassay will be one that uses affinity-purified PAbs or a set of MAbs at high dilution in an EIA format rather than an RIA one. In addition, the assay should demonstrate a LOD around 1 nM or better (much effort is still needed toward this goal). The assay will have to be validated using various matrices (e.g., herbivorous or carnivorous fish and *Gambierdiscus* spp. extracts) obtained through adequate clean-up procedures enabling near quantitative recovery. Moreover, the assay will need to be accurate and robust and ultimately portable *i.e.*, adaptable to field testing.

As a final requirement for definitive validation, the availability of sufficient amounts of specific Abs to CTXs will allow for inter-laboratory studies aiming to correlate immunoassay-generated data with conventional physico-chemical and more functional assays based upon biological or pharmacological properties of the CTX family. In any case, the assay must not yield false-positive results (negatively impacting the fisheries industry) or false-negative results (the worst from the public health point of view).

Methods must be actively pursued to standardize immunochemical reagents. For this purpose, long-term or inexhaustible sources of such tools and the issues associated with their use need to be correctly addressed, in order to define a routine cost-effective mass screening of fishes prior to consumption. Towards this goal, *in vitro* bioreactor systems (which enable adequate growth of hybridoma-producing specific MAbs) or genetically modified bacteria and plants (which can express Ab fragments of defined specificity and affinity) will be of great help.

Finally, it is noteworthy that the combination of MAbs 10C9 and 3D11 (directed to the left and the right wing of CTX-3C, respectively) was effective at neutralizing CTX-3C both *in vitro* and *in vivo*, protecting mice from an otherwise lethal injection of this toxin [207].

#### 3.3.5. Pharmacological assay: Receptor Binding Assay

A well established approach for the determination of CTXs, as well as other toxins acting on voltage gated sodium channel (VGSC), is the Receptor Binding Assay (RBA). RBAs are very sensitive and introduce a high degree of specificity. These have been developed since the 1980s from the isolation of VGSC obtained from animal tissues, and are basically implemented by competitive measures of radioactivity using tritiated saxitoxin (STX) or brevetoxin (PbTx) [183,232,233].

The VGSC contains multiple binding sites for several neurotoxins produced by diverse organisms such as marine dinoflagellates, brown algae, plants, scorpions, spiders, cone snails, sea anemones, corals, fishes and frogs [183,234,235]. The RBA is based upon the principle of the affinity of a given toxin for a specific binding site. The RBA measures the binding competition reaction between a radiolabeled toxin and a non-radiolabeled toxin that binds specifically to the common receptor of the ion channel.

Regarding marine toxins, VGSC inhibiting toxins like the saxitoxins (STXs) and tetrodotoxins (TTXs) target the VGSC binding site 1. RBA was applied to STX and TTX detection using tritiated STX as a binding competitor [236–243]. VGSC activating toxins like CTXs (P-CTXs, C-CTXs, I-CTXs) share a common receptor with brevetoxins (PbTxs), the binding site 5, which is located on the alpha subunit of the VGSC [95,108,109,146,148,191,243–247]. The binding activities of PbTxs and CTXs have been explored on brain, cardiac, and skeletal muscle sodium channel isoforms expressed in human, rat, marine mammals, marine turtles or fish neuronal tissues (for a review see Bottein Dechraoui *et al.* [191]). Comparative studies have shown that P-CTXs and C-CTXs are more potent to bind to site 5, with much higher affinity than PbTxs [33,146,148,188,246].

The RBA designed for CTXs traditionally measures the binding competition between ciguatoxin in the sample and a [^3^H]PbTx standard for the sodium channel receptor. The use of [^3^H]PbTx is a necessity, as presently no tritiated CTXs is available. This is due to the infinite quantity of CTXs present in biological samples and the large quantities (several μg) of pure toxins necessary for the synthesis of tritiated standards. Most authors cited above used [^3^H]PbTx-3 and a few used [^3^H]PbTx-9 [95,96,184,248,249]. [^3^H]PbTx-3 (15 Ci/mmol) is prepared by the reduction of PbTx-2 with [^3^H] sodium borohydride according to the method reported by Poli *et al.* [246]. Certified [^3^H]PbTx-3 is commercially available on demand and the purity of the [^3^H]PbTx-3 should be near 99% as determined by HPLC analysis and the stock solution should be kept at −80 °C. During the RBA, the concentration of [^3^H]PbTx-3 may vary between 0.5-3 nM according to authors.

The competitive experiment between CTXs and [^3^H]PbTx for receptor binding has been carried out in test tube [33,34,45,69,146,148,184,250–252] or microplate [95–97,147,188,253].

Originally, rat brain membranes were used in RBA as a source of VGSC. These are prepared essentially following the protocol described by Dodd *et al.* [254]. A protein assay is used to determine the total synaptosomes concentration of each preparation of rat brain membrane. In order to determine the optimal synaptosomes concentration to be used in the RBA, different volumes of the preparation are put in the presence of constant tritiated brevetoxin ([^3^H]PbTx) concentration. The concentration of synaptosomes used in the RBA varies from 40–150 μg mL^−1^ according to the authors [33,34,45,69,95–97,146,147,184,188,250–253]. Synaptosomes are stored at −80 °C for several weeks (up until six months).

The optimal binding conditions are checked with total binding giving no more than 10% of the total radioactivity and with non specific binding lower than 30% measured in the presence of saturating concentration of PbTx-3. Specific binding is obtained when subtracting non specific binding from the total binding. Saturation experiments are performed using constant synaptosomes concentration and increasing [^3^H]PbTx-3. Competitive experiments are conducted with synaptosomes concentrations chosen in the linear portion of the curve and constant [^3^H]PbTx-3 with increasing concentration of cold competitor (non radiolabeled toxin or sample to test). The bound and unbound cold competitor are further separated by centrifugation or filtration, and the amount of bound [^3^H]PbTx-3 is measured using a liquid scintillation counter in a given volume of scintillation cocktail (some scintillation cocktails are biodegradable allowing nonradioactive wastes). Then, each competitive experiment yields a sigmoidal dose-response curve with binding parameter data such as the concentration of cold competitor that induces 50% inhibitory radiolabeled binding *(IC_50_), Kd* (dissociation constant), *Ki* (inhibition constant), *Hill slope*, and *Bmax* (total binding sites).

When testing for biological samples, calibration of the assay is generally performed by testing each RBA with a known amount of purified toxins (PbTxs or CTXs) or with a toxic extract of *Gambierdiscus* with a known concentration of P-CTX or C-CTX equivalent [34,69,148]. Practically, the IC_50_ value for the biological samples is expressed in equivalent of cells (cell mL^−1^) for microalgae samples or gram of tissue (g mL^−1^) for fish samples. The IC_50_ value obtained with standard toxin or calibrated sample is substituted by the IC_50_ obtained with the biological sample for the quantification of toxins expressed in g PbTx, P-CTX or C-CTX equivalents per gram of fish tissue or per cell for algal samples [33,34,69,95,147,148,249]. For example, RBA toxicity could be expressed as pg P-CTX-3C eqv/cell for *Gambierdiscus* samples, and as ng C-CTX-1 eqv g^−1^ or ng P-CTX-3C eqv g^−1^ of flesh for fish tissues [33,34,69,148].

When testing biological sample for specific toxins, as for any detection method, the matrix effect has to be taken into consideration. This is because the matrix is likely to contain components that may interfer with RBA by increasing non specific [^3^H]PbTx-3 binding. These components could interact with the synaptosome membranes and disrupt the brevetoxin binding site on the sodium channel by altering protein structure or the interaction of the protein with other membrane component [250]. An increasing quantity of a known non-toxic sample is tested in the RBA competitive experiment to determine the maximum quantity of a sample that does not cause matrix interferences according to the protocol of preparation used. For each type of biological sample, the limit of quantification (LOQ) should be established. This will help establish the range of sample concentration or quantity that has to be tested. Regarding P-CTXs, the LOQ of the RBA was estimated to be 15.5 fg P-CTX-3C eqv cell^−1^ for algal samples and 0.155 ng P-CTX-3C eqv g^−1^ for fish samples [33].

The RBA was successfully applied for monitoring programs on ciguatera risk in three islands of French Polynesia: Tubuai, Raivavae and Nuku-Hiva [33,34]. Indeed, RBA proved to be a very valuable, suitable and sensitive tool for detecting ciguatoxins in all stages of the trophic chain of ciguatera *i.e.*, *Gambierdiscus* populations and fish from various trophic levels, thus providing early warning of increasing CFP toxicity in the trophic chain and in discriminating toxic areas from non-toxic ones. In particular, results showed that herbivorous fishes in these study islands could display RBA values as high as in carnivorous fishes and even higher [33,34]. Such data has important sanitary significance for local populations of the Australes archipelago, given the higher local availability of this trophic group in the Australes as compared to data from other archipelagoes [34].

Another common recommendation in CFP is to avoid the consumption of larger-size specimens [255,256]. However, in the case of these studies by Darius *et al.* [33] and Chinain *et al.* [34] no clear relationship was observed between RBA values and the size and weight of the 298 fish specimens tested in the Tubuai, Raivavae and Nuku-Hiva [33,34], suggesting that smaller-size specimens could be as dangerous as large ones.

The RBA tool also proved useful for identifying fish species with a high risk of ciguatera. As an example, based upon RBA values, Tubuai and Raivavae Islands were shown to share the same high-risk species, *i.e.*, *Scarus altipinnis,* with RBA values of 4.52 and 5.58 ng P-CTX3C eqv g^−1^ of flesh, respectively. On the contrary, the high-risk species in Nuku-Hiva was identified as *Crenimugil crenilabis*, for which maximum RBA values were found to be 2-3-fold higher than values recorded in Raivavae and Tubuai [33,34].

Finally, results yielded by RBA in Raivavae were found generally congruent with the local population’s knowledge regarding risky species and areas, suggesting that locally, the knowledge about ciguatera may not be scientifically complete but is functionally correct.

Regarding CFP risk management, absence of a clear clinically effective dose harmful to humans greatly hampers the definition of a regulatory threshold for RBA. Using the same approach as the one described by Lewis for P-CTX-1B and C-CTX-1 [18,19], the clinically effective dose of P-CTX-3C was estimated to be >0.33 and 0.31 ng eqv P-CTX-3C g^−1^ of flesh by Darius *et al.* [33], and Chinain *et al.* [34] respectively. Further RBA analyses were conducted on 11 leftover fish meals that had intoxicated consumers in Tahiti (French Polynesia). Symptoms experienced by patients included gastro-intestinal, neurological and cardiovascular disorders. Toxic samples displayed RBA values ranging from 0.38 to 2.69 ng P-CTX-3C eqv g^−1^ of flesh [257], suggesting the relevance of the proposed precautionary threshold of 0.31 ng P-CTX-3C eqv g^−1^ of flesh, especially with regards to individual susceptibility factors (e.g., heavy *vs*. occasional consumers, occurrence of previous CFP episodes, *etc.*).

Despite the relevance of RBA tools, the need for specific equipment and licensing requirement for the use of radiolabeled components may lead to obvious limitations in the application of this assay. Attempts to modify this assay to use label other than tritiated forms has led to the development of a competitive receptor binding assay based upon the use of brevetoxin-B2 labeled with a chemiluminescent acridinium moiety [193]. The acridinium brevetoxin-B2 seems to be a promising alternative to the conventional radioactive ligand in order to avoid constraints associated with RBA.

#### 3.3.6. Physico-chemical analysis: High performance liquid chromatography coupled with spectroscopic (UV, FLD) or spectrometric (MS/MS) methods

As it has been extensively described in Section 3.1, physico-chemical analysis of CTXs has involved HPLC since the 1960s, as purification methods were being developed for the isolation of the bioactive compounds (later described as CTXs) present in ciguateric fish and responsible for the intoxications [126,127]. The first approach implemented to purify these compounds - and which is still used during the extraction procedures and the HPLC analytical steps - followed general rules applied for the purification of natural substances. Basically, these methods included the use of liquid/liquid partitions, open chromatography and HPLC, all taking into consideration the polarity of compounds. We present herein a review of the last 20 years of the available protocols on the chemical analysis of CTXs, addressing both chromatographical issues and detection strategies.

##### 3.3.6.1. High-performance liquid chromatography with UV detection

Since most CTXs do not possess distinctive chromophor groups in their structures (e.g., series of alternating single and double bonds), they do not strongly absorb radiation over the UV/VIS region. As a consequence, detection of CTXs should be made at 210–215 nm, a poorly sensitive and non-selective range for detection. High-performance liquid chromatography coupled with UV detection (HPLC-UV) has been applied extensively as a strategy for the isolation of CTXs before its use in the characterization of CTXs from fish tissues and dinoflagellate extracts [29,45,94–96,99,102,134,135,197,258–260]. Legrand *et al.* [135] applied HPLC-UV with isocratic mobile phase as the final steps for isolation of CTX from viscera of moray eels (*Gymnothorax javanicus*). Separation involved two reversed-phase columns: LiChrosorb RP-18 column (4.6 × 250 mm, 10 and 5 μm) from Brownlee Lab and isocratic elution with different solvent mixtures methanol:water (80:20) or (90:10) and then with acetonitrile:water (65:35) [259]. Chromatography on samples of *G. toxicus* collected in French Polynesia was also reported on a Develosil ODS-7 column (8 × 250 mm, Nomura Chemicals) using a linear gradient starting from acetonitrile:water (85:15) to (100:0). Slight modifications of such conditions involved the use of an Asahipak ODP-50 column (column size not available) and isocratic elution with acetonitrile:water (75:25), which allowed isolation of up to 0.7 mg of P-CTX3C from 1,100 L of *G. toxicus* culture [99]. The same column was also applied for the isolation of two analogs of P-CTX3C, namely 2,3-dihydroxyCTX3C and 51-hydroxyCTX3C, by isocratic elution with acetonitrile:water (6:4) [197]. A similar approach was taken by Satake *et al.* [134] for the isolation of two additional toxins, firstly chromatographed together on a Capcell Pak C-8 (20 × 250 mm, Shiseido) with a linear gradient from 65 to 100% acetonitrile and then further separated individually on a Asahipak ODP-50 column with isocratic elution at 45 and 75% acetonitrile for each toxin, respectively.

The protocol applied for isolation of ciguatoxin-4A from *G. toxicus* cultures collected in Rangiroa Atoll (French Polynesia) followed a similar pattern. Isolation by HPLC-UV involved in this case an Asahipak ODP-50 column (10 × 250 mm, Showa Denko) and linear gradient elution with acetonitrile:water from (75:25) to (100:0), followed by chromatography in a Capcell pak C-8 column (4.6 × 150 mm, Shiseido) with acetonitrile:water (65:35) and finally in an Asahipak ODP-50 column with acetonitrile:water (85:15) [29]. Isolation of 2,3-dihydroxyCTX3C from moray eel viscera also used HPLC-UV in the last steps. Extract obtained after gel permeation chromatography was chromatographed several times on columns Develosil ODS-7 (10 × 250 mm) and Asahipak ODP-50 (10 × 250 mm) by linear gradient elution with acetonitrile:water from (60:40) to (100:0), then isocratically on a Asahipak ODP-50 Shodex column with acetonitrile:water (65:35) [260].

A simplified procedure was proposed for fractionation of dinoflagellate and fish extracts based upon a C8 silica-based reverse phase column (4.6 × 250 mm, 5 μm; Altech Assoc.) [258] using a linear gradient of methanol:water from (50:50) to (100:0) in 25 min. A standard mixture was injected between runs to check the stability of chromatographic conditions and for standardization of the retention times of the toxic fractions. The eluate was monitored by UV detection at 215 nm and the toxicity checked by MBA. The use of a C8 column instead of C18 seemed to be more adequate to achieve lower retention times and solvent consumption, especially for the less polar compounds that are usually present in dinoflagellate samples or herbivorous fish.

The isolation of P-CTX-1, -2 and -3 from moray eels was achieved similarly on reverse-phase columns LiChrocart (Merck) and polymeric PRP-1 (Hamilton) with isocratic elution with acetonitrile:water (50:50) for P-CTX-1 and (60:40) for P-CTX-2 and -3 [45]. Caribbean ciguatoxins C-CTX-1, C-CTX-2 and three other C-CTX-1-related compounds were isolated from the horseeye jack (*Caranx latus*) in a similar way with gel permeation columns, followed by PRP-1 (Hamilton) eluted with acetonitrile:water (1:1) and afterwards with LiChrocart C18 (Merck) eluted with acetonitrile:water (2:1) [94,102]. The eluate was likewise monitored by UV at 215 nm and toxicity checked by MBA as in previous works.

CTXs from the Indian Ocean (I-CTXs) were isolated from semi-purified extracts obtained by cleanup with florisil and size-exclusion fractionation with Sephadex and TSK columns. HPLC-UV was applied isocratically on a LiChrospher 100 RP-18 (4 × 250 mm, 5 μm; Merck) with methanol:water (90:10) at 1 mL min^−1^, then with methanol:water (88:12) in the same column and finally on a PRP-1 column (4 × 150 mm, Hamilton) eluted with acetonitrile:water:isopropanol (80:19:1). The eluant was monitored at 215 nm and toxicity tested by MBA and radiolabeled ligand binding assay (RBA) with tritiated brevetoxins (PbTx-3 or PbTx-9) [95,96].

In summary, the ultimate isolation of CTXs from pre-purified extracts obtained after liquid-liquid extraction, solid-phase extraction, and size-exclusion chromatography were achieved by reversed-phase HPLC-UV with C18, C8 and polymeric columns. Isocratic or linear gradients based mainly on acetonitrile:water binary systems ranged from 50:50 to 100:0 depending on the polarity of congener, which may be put in a hierarchical order according to the source (carnivorous > herbivorous > dinoflagellate).

##### 3.3.6.2. High-performance liquid chromatography with fluorescence detection

The presence of a primary hydroxy group at the side chain of the P-CTX-1 molecule suggested that the toxin could be derivatized into a fluorescent ester being suitable for high-performance liquid chromatography with fluorescence detection (HPLC-FLD). The strategy proposed by Goto *et al.* for derivatization of hydroxysteroids [261], based upon the commercially available reagent 1-anthroylnitrile (*i.e.,* anthrylcarbocyanide), was applied with this aim, achieving a detection level of 1 ng [262]. The coupling efficiency for the derivatization step was estimated at 81 ± 4% [263]. Since a primary hydroxyl group may be often present in compounds from such biological matrices, an additional clean-up was required after derivatization by solid-phase extraction with a Sep-pak silica cartridges (Millipore). The HPLC-FLD conditions were adopted from those previously proposed for analysis of pectenotoxin-1 [264], using isocratic elution with 85% acetonitrile on a Develosil ODS-5 (4.6 × 250 mm, Nomura Chemistry) column at 30 ºC and 1 mL min^−1^, and with the fluorescence detector set at 365 and 465 nm wavelengths for excitation and emission, respectively [92,265]. Such a procedure was successfully applied to samples of *G. toxicus* and to different carnivorous fish species, including *G. javanicus, Lutjanus bohar, Plectropomus leopardus* and *Epinephelus fuscoguttatus.* The applicability of this method was also demonstrated for P-CTX-2, P-CTX-3, 2,3-dihydroxyCTX3C; in this case changing the isocratic elution to acetonitrile:water (95:5) [17]. The method showed good linearity, which contributed to obtaining accurate results. Applying very similar conditions to 11 minor CTXs isolated from carnivorous fish demonstrated that some congeners lacked the primary hydroxyl group, and therefore did not derivatize (e.g., P-CTX3C). The derivatization protocol was also investigated using carbonyl azides or carbonyl nitriles of coumarin derivatives [266]; the reagent 7-diethylaminocoumarin-3-carbonyl azide provided the best quantum yields and fewer accompanying peaks in the chromatogram. The main advantage of using aminocoumarins instead of other reagents for derivatization is that a fluorescent chromophor and a nitrogen-containing moiety can be introduced in a single step, which enhanced the signal obtained in mass spectrometry analysis. HPLC-FLD detection was performed in this case at 238 and 455 nm for excitation and emission wavelengths, respectively, providing a limit of detection between 0.5–1.0 ng.

Another HPLC-FLD method was reported using continuous flow, post-column derivatization of the chromatographically separated toxins from *G. toxicus* [267]. Alkaline oxidation with periodic acid and ammonium hydroxide was used to form fluorescent derivatives in a similar way to that used for analysis of PSP toxins [268,269]. Nevertheless, the authors concluded that it was not possible to achieve quantifiable results using standard addition techniques, and analytical precision was much poorer than that obtained with the MBA. These facts may be explained by results from further separation by size exclusion chromatography, which revealed a molecular weight of toxic fraction around 38,000–40,000 Da. Such a difference suggests that they are unlikely to be related to CTXs.

In summary, despite the fact that HPLC-FLD methods are usually considered highly sensitive, the routine applicability to CTXs determination within monitoring programs is limited. CTXs are very potent toxins causing poisoning even at very low amounts. According to reported events, it was initially estimated that levels as low as 35 ng CTX/100g were toxic [17]. Taking into account the limit of detection of 0.5–1 ng on-column, such toxic levels might be achieved from a 50–100 g sample in the case that only P-CTX-1 is present. However, according to the safety limits recently reported of 0.01 ng g^−1^ for P-CTX-1 and 0.1 ng g^−1^ for C-CTX-1 [24], at least 50 and 500 g would be needed for analysis of C-CTX-1 and P-CTX-1, respectively. Additionally, at least 23 and 12 different congeners, which could contribute to the total toxicity responsible of the ciguatera fish poisoning, have been already described for P-CTXs [227] and C-CTXs [97], respectively. In carnivorous fish, it was estimated that nearly 60–80% of ciguatoxicity in MBA was related to CTX-1B (P-CTX-1), while 25% corresponds to other derivatizable CTXs [263]. Other congeners that lack the primary hydroxyl group may be present in samples, especially in herbivorous fish, making this approach unsuitable or uncertain to efficiently protect public health.

##### 3.3.6.3. High-performance liquid chromatography with mass spectrometry detection

The use of mass spectrometry (MS) for the analysis of ciguatoxins was already considered during the first stage of characterization and structural elucidation of congener structures, mainly by using fast atom bombardment (FAB) as an ionization technique. The introduction of ionspray (IS) as ion source for MS analysis of CTXs was reported by Lewis *et al.* in 1994 [270]. The variation on the ion source conditions applied, mainly the orifice potential led to measure the molecular and pseudomolecular ions of P-CTX-1 in different *m/z* intensities: 1,111.8 for [M + H]^+^, 1,133.8 for [M + Na]^+^, 1,129.8 for [M + H + H_2_O]^+^ and losses of up to five water molecules (*m/z:* 1,094, 1,076, 1,058, 1,040 and 1,022). The mass spectrometric analysis obtained with IS provided similar mass spectra and fragmentation patterns to those reported previously with FAB. The technique reached a limit of detection of 1 ng of pure CTX-1, equivalent to that reported for HPLC-FLD. However, severe ion suppression effects were found when a crude lipid extract of fish was spiked with P-CTX-1 at an equivalent of 1.5 ng g^−1^ flesh. Nevertheless, this was the first attempt to provide a simple, sensitive and confirmatory HPLC-MS coupled technique.

HPLC-MS analysis of highly purified extracts from the viscera of ciguateric moray eels (*Lycodontis javanicus*) allowed determination of up to 14 P-CTXs congeners at *m/z* of 1,095.7 (two), 1,111.6 (six) or 1,127.7 (six), including dominant ions for P-CTX-1, -2 and -3 [271]. In addition to the protonated species, each of these ciguatoxins gave rise to prominent ammonium [M + NH_4_]^+^ and sodium [M + Na]^+^ adducts. It was noted that acetonitrile:water gradient modified with 1 mM ammonium acetate provided better results than modification with 0.1% TFA, as well as the use of turbo-assisted HPLC-MS, which provided a 20-fold improved signal over conventional HPLC-MS. The limit of detection allowed easy detection of 4 ng g^−1^ P-CTX-1 in fish flesh. Adducts of sodium, ammonium, or even potassium, as well as ions resulting of up to five water losses have also been reported for C-CTXs [94]. This characteristic pattern proved to be very successful for identification of new CTXs congeners.

HPLC-MS was applied for the characterization of 12 C-CTXs accumulated by horse-eye jack (*Caranx latus*) that was previously purified with size-exclusion chromatography with a TSK column (Toyopearl) [97]. In this case, an HP 3300 HPLC pump was coupled with a hybrid quadrupole/time of flight analyzer API Qstar Pulsar (PE-Sciex). Ions corresponding to [M + NH_4_]^+^, [M + H]^+^ and [M + H − H_2_O]^+^ and [M + H − 2H_2_O]^+^ were present in significant intensities when formic acid was used as solvent modifier. On the contrary, lower intensities and absence of [M + NH_4_]^+^ pseudomolecular ion was obtained with TFA. Besides, formic acid also improved the base peak chromatogram and the MS spectrum by five-fold compared to TFA, and consequently seemed to be more adequate for CTX ionization. Reversed-phase separations were more efficient on a Zorbax C3 (2.1 × 150 mm, Agilent) than on an Aquapore C18 RP 300 (1 × 50 mm), especially to separate closely eluting congeners. Chromatographic gradient was performed with acetonitrile:water (both modified with 0.1% formic acid). The analysis of toxic fractions led to determination of five new CTX congeners at *m/z* for [M + H]^+^: 1,159.6, 1,143.6, 1,157.6, 1,127.6; plus several isomers of them and of the C-CTX-1, not previously reported. The same conditions allowed determination of some of these congeners in other fish species such as grey snapper (*Lutjanus griseus*), grouper (*Serranidae*) and a black jack (*Caranx lugubris*) [98], as well as in barracuda (*Sphyraena barracuda*), where ciguatoxins and also other hydroxyl-polyether compounds likely related to brevetoxins were identified [147].

I-CTXs were first reported by Hamilton *et al.* [96], isolated from red bass (*Lutjanus bohar*) and red emperor (*Lutjanus sebae*) fishes, and characterized by HPLC-MS in a similar way to previously reported for C-CTXs [97,98,147], with the exception of the chromatographic conditions. The conditions were modified to isocratic elution on a Zorbax 300SB (2.1 × 150 mm, 3.5 μm, Agilent) with acetonitrile:water:isopropanol (80:19:1) modified with 5 μM ammonium acetate or 0.1% formic acid. The molecular ion of I-CTX matched with that of C-CTX at *m/z* 1,141.6, though relative ion abundances among molecular and pseudomolecular ions differed, with the pseudomolecular ion [M + H - H_2_O]^+^ the most abundant for the Indian congener. Further studies on red emperor led to characterization of three additional Indian ciguatoxins (I-CTX-2 to -4). I-CTX-1 and -2 showed indistinguishable masses from C-CTX-1 and *ca.* 60% of the toxic potency of P-CTX-1. The spectra patterns involved the typically associated ions, namely [M + H]^+^, [M + Na]^+^, [M + NH_4_]^+^, [M + H - nH_2_O]^+^, with n from 1 to 3. It was presumed that I-CTX-1 and -2 may arise from different dinoflagellates and then oxidized to I-CTX-3 and -4.

A further work from Lewis *et al.* reported coupling of HPLC with tandem mass spectrometry (MS/MS) [272]. The MS/MS was tuned by direct infusion of pure CTX standard in 50% acetonitrile:0.1% aqueous TFA. Product ions for MRM transitions were optimized for the loss of NH_3_ from the dominant [M+NH_4_]^+^ pseudomolecular ion and additional losses of up to three water molecules. Thus, transitions were (*m/z*): 1,128.7 > 1,094.0/1,076.0/1,058.0 for P-CTX-1 and 1,158.6 > 1,123.6/1,105.6 for C-CTX-1. The method provided a linear response within the working range and improved sensitivity: the limits of detection were as low as 0.04 and 0.1 ng g^−1^ fish flesh for P-CTX-1 and C-CTX-1, respectively. Chromatographic conditions used a reversed-phase column Vydac (2.1 × 250 mm, 5 μm; Separation Group) eluted with linear gradient acetonitrile:water modified with 0.05% TFA.

An 1100 HPLC (Agilent) coupled to a 4000 QTRAP hybrid mass spectrometer (Applied Biosystems) was applied for the confirmation of P-CTX-1 and C-CTX-1 on the basis of mass and retention times compared with reference materials [119]. Working under triple quadrupole mass spectrometry basis (LC-MS/MS), a highly specific detection was achieved. For C-CTX-1, three MRM transitions were selected using the pseudomolecular ion [M+H−H_2_O]^+^ as precursor ion (*m/z*: 1,123.5 > 1,105.6/1,087.6/1,069.6), and the response added. For P-CTX-1, two groups of transitions were monitored and the response added. The first took the molecular ion [M + H]^+^ as precursor ion (*m/z*: 1,111.6 > 1,093.6/1,075.6/1,057.6) and the second was based upon the ammonium adduct [M+NH_4_]^+^ (*m/z*: 1,128.6 > 1,093.6/1,075.6/1,057.6). Turbo ion spray conditions were identical for nebulizer gas (50 psi), turbo gas (50 psi), temperature (400 ºC), curtain gas (20 psi), and IS voltage (+5 kV). Collision energy was specifically optimized for each transition and ranged between 15–37 eV. Chromatographic conditions used reversed-phase Luna C8(2) column (2 × 150 mm, 5 μm; Phenomenex) and programmed linear gradient with acetonitrile:water modified with 0.1% formic acid. This procedure was successfully applied, in combination with cell-based assays, as a confirmatory technique in the study of CFP outbreaks since 1999.

**Figure 8:**
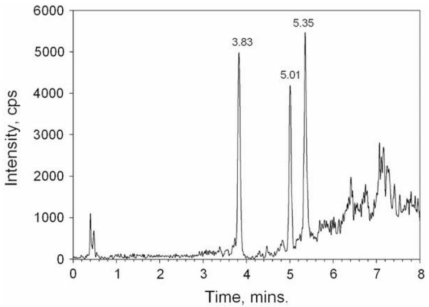
Total ion chromatogram for a fish sample extract containing P-CTX-1 (3.83 min, 0.8 mg/kg), P-CTX-2 (5.01 min, 1.1 mg/kg) and P-CTX-3 (5.35 min, 1.4 mg/kg). Reprinted from [125], reprinted with permission from Elsevier.

Recently, improved HPLC-MS/MS methods have been reported by Australian groups. Lewis *et al.* [124] reported HPLC-MS/MS analysis following a ciguatoxin rapid extraction method from 2 g fish flesh sample. A limit of quantification of 0.1 ng g^−1^ was achieved, which was suitable to confirm suspect ciguateric fish in the Pacific Ocean [124]. In this case, the HPLC system was equipped with a Luna C18 column (2.1 × 250 mm, 5 μm) and a guard column (4 × 2.1 mm, 5 μm), both from Phenomenex. The program gradient was performed with a binary mobile phase system consisting on 95%acetonitrile:water modified with 2 mM ammonium formate and 0.1% formic acid. The MS/MS systems were triple quadrupole API 2000 and API 4000 QTRAP (Applied Biosystems), the latter more sensitive. The three MRM transition pairs for P-CTX-1 were based upon the fragmentation of the pseudomolecular ion [M + NH_4_]^+^, with subsequent loss of ammonia and successive losses of water (*m/z*: 1,128.7 > 1,093.7/1,075.7/1,057.7). Soon after, Stewart *et al.* presented a modification of the rapid extraction method proposed by Lewis *et al.* with HPLC-MS/MS analysis of P-CTX-1, -2 and, -3 in ciguatera-suspect fish [125] (Figure 8). The chromatographic system consisted of Prominence LC (Shimadzu Corp.) equipped with a Gemini C6-phenyl column (2 × 50 mm; Phenomenex) and an AB/Sciex API4000Q (AB/MSD Sciex). Acetonitrile:water-based gradient, similar to other previously reported and equally modified with 2 mM ammonium acetate and 0.1% formic acid, was used. The MRM transitions for MS/MS detection were based upon those previously reported [124], and extended to the analysis of CTX-2 and -3 (*m/z*: 1,128.7 > 1,093.7/1,075.7/1,057.7 for P-CTX1 and 1,112.7 > 1,077.6/1,059.6/1,041.6 for P-CTX-2 and -3).

Similar conditions to previous work for HPLC-MS [96,97] and for HPLC-MS/MS [124] were applied for confirmation of P-CTXs as causative agents in samples of toxic fish and *post-mortem* human liver after a fatal human poisoning [273].

Several works have focused on the analysis of CTXs present in dinoflagellate cultures. Apart from those previously mentioned that focused on isolation and characterization of congeners using HPLC and further FAB-MS off-line, the development of coupled HPLC-MS/MS methods have motivated further studies on toxin production by species of the genus *Gambierdiscus*.

Roeder *et al.* [122] conducted a series of studies on cultured strains of *Gambierdiscus* from different origins. In this case, chromatographic separation was performed on Shimadzu HPLC system with Hyperclone C8 column (2 × 50, 3 μm) and guard column, both from Phenomenex. Separation was achieved with a gradient elution program of acetonitrile:water modified with 5 mM ammonium formate. MS/MS detection was carried out by an API 365 MS/MS triple quadrupole (Applied Biosystems) with Turbo Ion Spray as ion source. MRM transitions were acquired in this case both in positive and negative mode. To the best of our knowledge, this is the only work in which CTX determination has been performed in negative mode. According to the authors, the application of negative polarity provided even more sensitivity than the positive one. However, due to the lack of proper analytical standards, additional toxicological or functional studies should have been performed on LC-fractions to confirm their activity.

Ciguatoxins from cell pellets obtained by culturing several strains of *Gambierdiscus* isolated from Rarotonga lagoons were analyzed using an UPLC Acquity system (Waters Corp.) equipped with a BEH-C18 column (50 × 1 mm) packed with 1.7 μm particle size (Waters) and coupled to a Quattro Premier triple quadrupole MS (Waters). The mobile phase consisted of a linear gradient from water to acetonitrile with 0.1% formic acid. The analysis was performed in positive mode for congeners CTX-3B, -3C, -4A and -4B. Multiple Reaction Monitoring (MRM) transitions monitored molecular ions [M + H]^+^ for precursor ions and then the fragments at *m/z* 165 and 125 for product ions, according to the product ion spectra obtained intra-laboratory. The use of such alternative product ions indicates that the maximum sensitive and selective in MRM analysis should be investigated for each type of instrumentation by tuning the MS with proper CTXs standards. The advantages of using ultra-performance chromatography and separation on sub-2 μm particle size columns include enhanced efficiency, sensitivity and high-throughput analysis. In this case, retention times ranged from 3.45 to 4.00 min for the congeners analyzed, and the limit of detection that was estimated with non-certified standard solution was 10 pg loaded on column [70]. None of these congeners were detected in the cultured strains, even though they showed toxicity to Neuro-2A cells.

The production of CTXs by several strains was also investigated for different species of *Gambierdicus* isolated in French Polynesia by MBA, RBA and HPLC-MS/MS [69]. After purification and fractionation by low and high pressure liquid chromatography, HPLC-MS analyses were conducted on an Agilent 1100 HPLC equipped with a Luna C18(2) (2 × 50 mm, 5 μm; Phenomenex). The elution was isocratic 70 or 80% acetonitrile modified with 0.1% or 0.3% acetic acid; and delivered to an MSD Sciex API 4000 triple quadrupole (Applied Biosystems). Interestingly, to the best of our knowledge, this is the only report in which two alternative ion sources (Turbo Ion Spray, TIS and Atmosphere Pressure Chemical Ionization, APCI) were tested. APCI was shown to be more efficient in promoting the molecular ion [M + H]^+^ and avoiding [M + Na]^+^ adducts. A wide variety of congeners were determined, though P-CTX-3C, -3B, -4A, -4B and M-seco-CTX-3C added up to 97–98% of the total CTXs found in two *G. polynesiensis* clones. Interestingly, the toxin profiles seemed to be characteristic for each clone and their total toxin content increased with the age of culture.

During the course of this study, a potential mistake on the molecular ion associated to P-CTX-3C firstly reported by Satake *et al.* [99] has been identified. Several publications and reviews have assigned an *m/z* value for molecular ion [M + H]^+^ of 1,045 for P-CTX-3C [20,122,275,276]. However, according to the original reference [99], this *m/z* value seems more likely to be that of the sodium adduct [M + Na]^+^, which can be promoted in the analysis of algal samples with relatively high salt content.

In summary, HPLC-MS and HPLC-MS/MS have proved to be extremely useful for the identification and characterization of new CTXs congeners around the world. Moreover, forefront instrumentation available over recent years has provided enhanced sensitivity and selectivity by measuring accurate masses or a series of fragment ions according to characteristic patterns. Such advances have opened a new era of application of mass spectrometric detection methods for unequivocal confirmation of ciguatera suspect-fish. However, taking into account the safety limits recently reported (0.01 ng g^−1^ and 0.1 ng g^−1^ for P-CTXs and C-CTXs, respectively [18,19,24,119,120]), it seems that HPLC-MS methods are still limited when needed for accurate quantitative results from very close borderline samples. Improvements in instrumentation and in sample preparation will allow the routine application of HPLC-MS to overcome some matrix effects from fish samples and decrease the limits of detection and quantification. Additionally, combination with a screening technique may also provide improvements in sample throughput.

## 4. Perspectives to Confront the Onset of Ciguatera in Europe

### 4.1. Ciguatera in Europe

Cases of CFP in Europe are usually associated to ciguateric seafood imported from ciguatera-endemic areas or to CFP in travelers returning from endemic CFP areas [5–8]. In 2005, Perez-Arellano *et al.* [9] reported for the first time the occurrence of CFP in the Canary Archipelago (Macaronesia). In January 2004, a 26-kg amberjack (*Seriola rivoliana*) was captured along the Coast of the Canary Island, filleted and consumed by five family members. Symptoms appeared between 30 minutes and 28 h after consumption and resolved after several weeks, but recurred intermittently with a lower intensity for several months. The symptoms exhibited were gastrointestinal, neurological, cardiovascular and general symptoms typical of CFP such as reversal of hot and cold sensation, though the affected family members did not require hospitalization [9]. Analysis of the fish portion gave positive results using an immunobead assay (not identified) and presence of CTXs was confirmed using Neuro-2a cell based assay as well as LC-MS/MS analysis. CTX content was low and estimated at 1.0 ng g^−1^. Caribbean CTX-1 was identified by LC-MS/MS in addition to two non-identified CTX-like compounds [9]. This case of CFP was the closest confirmed case occuring to Europe with evidenced presence of CTXs (LC-MS/MS) and the first case of intoxication from temperate waters of the Eastern Atlantic Ocean.

Epidemiological data have reported in the past various cases of ichtysarchotoxism in the Eastern Mediterranean Sea (Israel coasts) after consumption of rabbitfish (*Siganus luridus*) in 1971–1972 [277] and in 2002 [278], saupe (*Sarpa salpa*) in 1988 [279] and other edible fish (*Siganus* spp.) in 2004–2007 [280,281] which were tentatively described as CFP. In the latter case [281], a membrane immunobead assay tested on various fish samples gave a positive response, but the presence of CTXs was not confirmed using RBA, chemical or toxicological studies [281]. Moreover, misinterpretation of the CFP-induced saupe consumption [279] with *Caulerpa* poisoning was proposed by Chevaldonné [37]. De Haro and Pommier [282] recently distinguished CFP from ichthyoallyeinotoxism caused from consumption of saupe that is probably associated to Caulerpaceae [37]. Possible misdiagnoses of CFP with other forms of ichthyosarchotoxism have raised the necessity to confirm the presence of CTXs in the food or blood of affected patients in order to confirm the occurrence of CFP within these areas.

Recent epidemiological data reported two outbreaks of ichthyosarchotoxism diagnosed as CFP, which occurred in 2007 and 2008 in Madeira Archipelago (Eastern Atlantic Ocean) 260 miles north of the Canary Islands Archipelago [10]. In the first case, a total of six patients were intoxicated after consumption of various fish species *i.e., Seriola* sp., *Sparisoma credence*, *Serranus atricauda*, *Bodianus scrofa*, *Balistes capriscus* and *Pagrus pagrus* captured at the Salvage Islands. Neurological symptoms lasted between 0.5 and 1.5 months. The second case concerned intoxication of 11 crew-members after the consumption of a 30 kg fish of the genus *Seriola*, captured at the Big Salvage Island. Neurological and gastrointestinal symptoms, lasting for one month, appeared as early as four hours after fish ingestion and required hospitalization of affected people. Presence of CTXs could not be confirmed in the fish flesh. In 2009, a 71 kg *Seriola dumerili* was fished at the Salvage Islands and one portion was tested using the “Cigua-Check Fish poison Test”(Oceanit). The immunoassay tested positive, but presence of CTX-like compounds has not yet been confirmed using chemical, pharmaceutical nor toxicological methods [10].

More recently, among a total of 64 samples collected along the coast of Cameroon, three were tested toxic using the Neuro-2a CBA [151]. This time, presence of CTXs compounds in toxic samples was further supported by LC-MS analysis, confirming a ciguatera outbreak along the coast of the Eastern Atlantic Ocean.

### 4.2. *Gambierdiscus* species in the Mediterranean Sea and Macaronesian waters (Canary Islands)

In 1948, a dinoflagellate reported as *Goniodoma* sp. [283] was detected in a sample collected from Boa Vista Island (Cabo Verde). The detailed description given for this dinoflagellate coincides very well with the original description of *Gambierdiscus toxicus* published almost two decades later [28]. Therefore it is likely that Silva’s description of *Goniodoma* sp may actually constitute the first evidence of the presence of the genus *Gambierdiscus* in Eastern Atlantic Ocean waters. In 2004, Fraga *et al.* reported for the first time the presence of *Gambierdiscus* sp. in the Canary Islands, at a latitude 12º N of Cabo Verde [15]. Based upon the recent revision of the taxonomy of the genus *Gambierdiscus* by Litaker *et al.* [73], morphological and molecular analyses are currently in progress on *Gambierdiscus* clones isolated from the Canary Islands, in order to specify whether these Eastern Atlantic clones should be placed in a separate species group. Regarding the toxicity of the Canary Islands isolates, recent studies conducted on *Gambierdiscus* cells crude extracts revealed high unspecific toxicity on the Neuro-2a CBA [192], probably associated to a high MTX-like activity [137,284]. No data are currently available regarding CTX production.

As the first description of *Gambierdiscus* spp. in the Canary Islands waters also coincided in time with both ciguatera outbreaks reported, the issue of a possible impact of *Gambierdiscus* blooms on public health and local fisheries in this area should also be considered. So far, there is no direct proof that these events are related, as amberjack can migrate and could become toxic far from the Canary Islands where it was captured. Moreover, other groups of benthic dinoflagellates such as *Ostreopsis*, *Coolia*, and *Amphidinium* commonly present in ciguateric biotopes were also detected epiphytically on benthic macroalgae in Tenerife, along with *Gambierdiscus* cells. Very recently, additional strains of a yet undetermined but different *Gambierdiscus* species have been isolated in the Canary Islands [285].

This same observation also applies to *Gambierdiscus* populations reported in 2007 in Crete Island (Greece) [14], which constitutes the first record of these genus in the Mediterranean Sea. This finding conduced the re-examination of archival material from Crete Island, and after additional study of fixed samples *Gambierdiscus* spp. could be confirmed as present since 2003 [13]. Although the morphological descriptions conducted at that time on this material led to the conclusion that it fitted well with the “*G. toxicus* type” as defined by Richlen *et al.* [72], it was not assigned to a species, as the taxonomy of the genus remained unclear [13]. Morphological and molecular analyses are currently underway in the light of the newly revised taxonomy of this genus [73] in an attempt to clarify the identity and the origin of these Cretan isolates. As for their toxic potential, preliminary toxicological studies based upon the Neuro2a-CBA revealed the lack of production of MTX-like compounds in *in vitro* condition*s* [284]. They also tested negative with MBA [12]. These data are consistent with the observation that the presence of *Gambierdiscus* in Crete has not coincided nor has been associated with the occurrence of CFP cases since 2003. However, it raises questions about a potential risk of CFP within this area, and to a larger extent within the Mediterranean Sea.

The identification of tropical and subtropical representatives of potentially toxic benthic dinoflagellate genera, such as *Ostreopsis* and *Gambierdiscus*, in the Eastern Atlantic Ocean has posed the question of the impact of climate change on the spreading of microalgae to more temperate waters [286]. This hypothesis is in accordance with the “tropicalization scenario” of the Mediterranean Sea proposed by Bianchi [287], taking also into account the increase of sea surface temperature [288]. The detection of *Gambierdiscus* spp. for the first time in the Mediterranean Sea [14], along with the increasing records of blooms of *Ostreopsis* spp. in the area [11,12,289,290–293], are consistent with the aforementioned hypotheses. As in the case of other aquatic organisms, the presence of *Gambierdiscus* in the Eastern Mediterranean Sea raises the issue of its origin; lessepsian migration (through the Suez Canal) or invasion via the Strait of Gibraltar are general possible hypotheses, however, further phylogenetical, taxonomical and toxicological analyses will shed light on *Gambierdiscus* phylogeography. Anthropogenic introduction of the species cannot be excluded, although there is no evidence or objective observation that may suggest such an origin. The fact that ciguatoxins from the Pacific are different from those from the Caribbean [94–98] might be an indication that since *Gambierdiscus* and *Ostreopsis* share similar ecology, these genera may have similar biogeographical patterns. Distinct biogeographical patterns are observed among *Ostreopsis* species from the Indo-Pacific Ocean and those of the Atlantic Ocean including the Mediterranean Sea [294]; the two warm water realms that became separated as a consequence of the closure of the Panama Isthmus. Despite accumulating evidence, the possibility that increased records may, in part, reflect an intense research in the field of marine benthic dinoflagellates during the last years should not be neglected [14]. In respect to the distribution of *Gambierdiscus* spp. and possible risk associated to ciguatera, one may not forget that toxicity studies on several *Gambierdiscus* strains of different latitudinal distribution in the western Atlantic showed that in the Northern hemisphere, *Gambierdiscus* strains located at higher latitudes (Bahamas) were less toxic than those at lower latitudes (Caribbean) [295].

There is increasing discussion about the link between climate variability and harmful algal blooms, (*i.e.*, geographical expansion of toxic benthic dinoflagellates, as a possible consequence of climate change), and its impact on human health (e.g., ciguatera, palytoxin-like poisonings) [11,78,296–298]. A survey in the South Pacific [78] particularly aimed to verify the link between ciguatera and climate change. This study suggests a model that contrasts the simple link between sea surface temperature increments with occurrence of CFP, although sea surface conditions need to be favorable long enough to generate enough load of ciguatoxins in ecosystems for occurrence of CFP in human populations. Moreover, accumulation, metabolism and detoxification kinetics may be altered in fish vectors in response to variation of ambient temperature, as described for tetrodotoxins [299] or organic pollutants [300]. Diversity of fish species as toxin vectors may also be altered in response to different climatic conditions [301]. Global warming is the explanation for the increasing reports of tropical fishes at higher latitudes [302]. Some of these fishes, which become toxic in ciguateric areas, could expand CFP to higher latitudes by their migration, although *Gambierdiscus* spp. is absent from these areas. Extrapolation of this model to the Mediterranean and Macaronesian waters suggest the importance of monitoring sea surface temperature in addition to *Gambierdiscus* spp. populations for the assessment of a possible onset of ciguatera in the Mediterranean Sea and Macaronesia in relation to global warming.

### 4.3. EU regulation for CTXs

The presence of marine biotoxins in seafood products is regulated in order to assess the consumer’s safety. European regulations enforce specific hygiene rules for food of animal origin, including toxins, affecting human health [160]. For CFP, this regulation states that “Fishery products containing biotoxins such as ciguatoxin or muscle-paralysing toxins must not be placed on the market”. This text was further extended to “ciguatoxins or other toxins dangerous to human health”[303]. Regulations EC No 853/2004 [160] and EC No 1021/2008 [303] also targets other types of ichtyosarcotoxisms (e.g., “fishery products derived from poisonous fish of the following families are not placed on the market: *Tetraodontidae, Molidae, Diodontidae* and *Canthigasteridae”*). These regulations also consider marine toxins present in shellfish products and set the maximum permitted levels of some toxins present in shellfish products. For shellfish toxins, contrarily to ciguatoxins, European regulations clearly indicate the reference analytical methods for their detection [304]. These official methods include the mouse bioassay, chromatographic methods and immunosorbant assay [304–306] and may open the use of LC-MS/MS and other methodologies in case of validation. In addition to the monitoring of biotoxins in fishing products, the presence of toxin-producing phytoplankton is also routinely monitored in order to fulfil European legislations [307].

As previously emphasized, European regulations give no indication about a reference analysis method for CTXs, nor about regulatory safety limits in fishery products. This lack of specification should not be interpreted as a lack of interest, as several countries in Europe—e.g., France, The United Kingdom, The Netherlands—actually have territories in tropical and sub-tropical ciguatera endemic areas periodically affected by ciguatera. Instead, this might reflect the difficulties that laboratories dealing with ciguatera have encoutered during the last decades regarding the definition and standardization of CTX determination methods and establishment of safety limits. As for the tentative limits proposed by several authors cited herein [18,19,24,119,120] and the comprehensive evaluation of methods for CTXs detection provided in this review and elsewhere [7,19,24,119,308], we expect that the EU will soon favor a scientific and technical coordinated effort in order to clarify these issues.

The European Food Safety Authority (EFSA) is currently completing a mandate to issue a scientific opinion on ciguatoxins (Mandate M2006-0060 on Emerging toxins-ciguatoxins) in response to a question raised by the European Commission (DG-SANCO). The mandate is expected to be completed by July 2010.

Of note, within the EU, national regulations specifically addressing CFP issue do exist in some countries. For example, some French overseas departments and territories are mainly located in ciguatera endemic areas (French Polynesia, La Reunion, Martinique). The French legislation directly incorporated the European directive, which is applicable for products imported into France from outside the EU and permits the import of certain marine species off a positive list [309]. In French Polynesia, groupers, snappers, barracuda and surgeonfish are banned for sale [310].

### 4.4. A risk analysis approach to confront CFP

The risk analysis approach is highly advisable to face CFP and establish recommendations when dealing with CFP and public safety. As for any other food intoxication, it is also very important that CFP risk analysis is coordinated by one agency or body, even if several agencies or people have to be involved.

In the process of risk analysis, three major issues should be addressed: risk assessment, risk management and risk communication. Numerous reviews on CFP risk, but also addressing other CFP issues, have contributed to CFP risk assessment [3,7,18,19,23,24,255,311,312]. Secondly, risk management should propose actions aiming at the reduction of risk, mainly through the establishment of monitoring programs for CTX producing species and toxins in food. Risk management should also set-up of complementary actions intended for the mitigation of ciguatera impact, and ideally reduce the risk. Examples of complementary actions could include providing general guidance for consumers, controlling imports and improving medical assistance. Finally, risk communication should complement these two steps to modulate “population behavior” in ciguatera endemic regions and improve communication with competent agencies having links with food safety and medical care. With this strategy in mind, one may face the difficulty of assessment and management of ciguatera risks and establish sounded communication policies. After these initial remarks, which constitute a simplification of the strategy proposed, one may get into more detail to identify key actions to establish a thorough program to face CFP risk analysis.

#### 4.4.1. CFP risk assessment

The different steps regarding risk assessment should consider hazard identification, hazard characterization, exposure assessment and risk characterization. For CFP, efforts should concentrate on CTXs identification, toxicity evaluation and epidemiology.

A recent review on ciguatera fish poisoning and its’ treatment, prevention and management [23] resumes the key actions to undertake, mainly through the determination of CTXs in fish, the evaluation of symptoms, collection of epidemiological data and treatment of CFP. The authors propose, as preventive actions, fish avoidance recommendations and communication strategies. However, in addition to the objective of risk identification of ciguatera in a given region, additional information is certainly of interest for risk assessment studies: such examples include the characterization and distribution of the potentially ciguatoxicogenic microalgal species, as has been reported elsewhere [33], the distribution of fish populations at risk, ethnological surveys regarding local remedies and avoidance strategies [34]. It is therefore understandable that a systematic approach is needed regarding the different factors that may contribute to understanding CFP in a given area, in order to have the widest assessment of CFP risk. Organizing the different levels at which we may identify risk factors influencing CFP can contribute to facilitate this systematic approach (Table 3).

#### 4.4.2. CFP risk management

Risk management is often presented in four steps, which include risk evaluation, risk management option assessment, implementation of management decisions and monitoring and review of management actions. As for the risk management of CFP, different countries or regions affected have developed through the years several options and decision-making protocols that are regularly implemented [23]. CFP management relies on different actions (Table 4), which include having a sound and updated legislation, an appropriate monitoring program for the identification of toxins with reliable laboratory tools (focusing on fish, microalgae and environmental parameters), a good system for the identification, treatment and reporting of CFP cases, as well as excellent coordination and communication between agencies implicated. The management of CFP is a response for the protection of both the public health and the local industry (mainly fisheries and tourism). Several important issues involved in CFP have to be taken in consideration.

The large number of fish species involved in CFP toxin transmission and the high degree of variability in toxin content within a given species is one of the major limitations for CFP monitoring. As a matter of fact, fish catches in CFP endemic areas are often recreational or in the hands of local fishermen working independently. This situation noticeably contrasts with shellfish products, for which most production areas are well defined in space and information provided to the producer and control of the production is easier. The availability of simple, fast and reliable tests that could be applied efficiently for the immediate screening of all individual fish on shore or in the market still needs to be achieved. Indeed, most methodologies for CFP toxin determination rely on sophisticated protocols which require long and elaborated laboratory manipulations as well as well-equipped laboratories.

Moreover, the density of *Gambierdiscus* spp. populations within a given area and their toxin production could determine the eventual risk of CFP in the area. However, the fact that the CFP toxin trans-vector (*i.e.*, fish) are mobile, and the fact that toxin in fish could result from a long-term accumulation, their toxin content may not be representative of the concentration of microalgae at the time and place where the fish was caught. It is therefore advisable to invest management efforts both in preventive and palliative measures.

As a response to the onset of CFP in Europe, it will be important to take in consideration various issues for the efficient management of CFP. Studies related to the development of toxin detection techniques and to the presence, distribution and toxicity of toxin-producing species and toxic fish are required on a long-term basis, provided that CTX standards and CTX reference materials are made available. The resulting information may identify species and fishing areas with high risk of CFP. Epidemiological data are clear indicators of the hazards of CFP within a given area. It is therefore important to favor the report of CFP cases to public health professionals and to confirm the presence of CTXs in leftovers meals, in order to facilitate differential diagnosis with other forms of seafood poisoning or fish intoxications (ichthyosarcotoxaemias). Additional effort towards the development of epidemiological studies is therefore advisable. Ethnological studies and surveys can also reveal valuable information for risk management actions and risk assessment. It is important to state that the geographical distribution of CFP is not only determined by the presence of *G. toxicus* and toxic fish, but also by anthropological considerations such as fishing and food habits in the population, local control and regulations. Surveys among the population can establish tawareness of CFP within a given area and bring to the surface interesting information such as the identification of the most dangerous fish for CFP within the area.

As for the treatment of CFP, reported in Section 2.2, mannitol administration (as well as other symptomatic and supportive treatments) has been widely described; a comprehensive synthesis is presented by Friedman *et al.* [23]. Administration of mannitol early after intoxication seems the most efficient palliative medical treatment. Medical centers in newly-affected areas should be aware of the risk of CFP and of the efficiency of mannitol and other treatments. Additionally, local traditional medicine is not to be ruled out, as it may be also effective [60,62,313].

#### 4.4.3. CFP risk communication

Risk communication strategy is a matter of serious concern: it has major impact on the different bodies and people involved, and influences the fate of hazards. A primary requirement regarding risk communication of food hazards is that the body in charge of disseminating the information regarding the risk has to be well acquainted with the exhaustive and updated scientific and technical information involved. A second issue is to define the strategy(ies) of risk communication chosen, which involves determining several factors such as the objective for such communication, the targets and the existing communication tools adapted for the situation. Risk communication has to take into consideration and predict the perception and reaction of the end-users of the message: consumers, other agencies, medical bodies, food industry, *etc*.

CFP is a complex phenomenon and the risk communication strategy for CFP is far from evident (Table 5). On the one hand, the complex transfer of toxins through the food webs and fish species involved does not always allow a clear determination of which fish species should be avoided. On the other hand, difficulties to estimate CTX content in food on a large scale have apparently not been overcome by technical advances of methods. Having these two examples in mind, one may consider that the risk communication strategy for CFP may be designed considering limitations regarding technical and scientific knowledge. Since CFP may result from the introduction of ciguateric fish into the market by numerous people implicated in fishing and processing of fish, issues on risk communication for ciguatera - basically oriented towards avoidance of fish according to species, size or origin - cannot be selectively targeted. Instead, risk communication may need to be widespread to reach all consumers and fish traders. Informative campaigns in the population should be designed in order to improve safer practices regarding fish food habits that could support local regulations. Information to visitors on advisable food habits, species of fish and areas at risk is also required. CFP risk communication on the other hand may have to be constant or iterative, avoiding the creation of a communication vacuum, but also having in mind the possible consequences of an amplification of risk perception. Ciguatera risk communication may also be targeted selectively to agencies or specific bodies (retailer associations, medical staff, tourist corporations, *etc.*) as a preventive measure to reduce cases of CFP intoxications: this is especially a very important issue in non-ciguatera endemic areas with presence of CFP caused by ciguatoxic fish imports or people travelling to ciguatera endemic areas. Selective targeted CFP risk communication can also favor transfer of information among agencies to improve CFP risk assessment, such as obtaining more accurate and reliable epidemiological data.

### 4.5. Application of risk analysis for CFP in Europe

Risk analysis studies are elaborate, require skilled working groups and may be costly. Considering the current state of CFP in Europe, one may question the relevance of setting up a risk analysis program in Europe. Three pieces of evidence contribute to the unequivocal settlement of CFP as a health hazard in Europe: (i) reports of CFP intoxication cases in European hospital and other medical institutions linked to fish imported from the tropics or to travelers returning from ciguatera areas; (ii) presence of fish containing CTXs in the Canary Islands and suspicion of toxic fish in Madeira archipelago; and (iii) recent reports of the ciguatera-causing dinoflagellate *Gambierdiscus* in the Canary Islands and in the Mediterranean Sea (Greece).

At this stage, it is difficult to define the current awareness of the administration in Europe regarding CFP, and one may postulate that European countries having territories in CFP endemic areas, such as France or the Netherlands, may be better acquainted. As previously outlined, European legislation takes into consideration CFP, but does not provide any elaborate information regarding the definition of toxins levels and methods for toxin determination.

Having this evidence in hand, and being aware that it is also important not to magnify the risk perception associated with the consumption of fishery products, risk analysis for CFP in Europe is a necessity. This is particularly apparent when we take into consideration that travelling to tropical areas is not anecdotic and that the distribution of ciguateric fish and CTX-producing microalgae might be expanding in European waters. Risk analysis for ciguatera in Europe should be addressed by implementing efforts proportional to the present situation, maintaining the commitment to assure an extensive assessment of the current CFP situation in Europe in order to correctly define the management and communication strategies that should be developed.

It may be expected that a risk analysis study on CFP to define detailed risk assessment and management plans as well as establish a proportionate risk communication strategy, may be considered in the future. Some preliminary and basic recommendations at this stage on possible priority actions needed in Europe are presented: Preliminary needs:

- Favor that the competent authority will lead actions related to risk analysis for CFP, in coordination with the different agencies implicated.
- Identify research agencies implicated in CFP to improve risk analysis.
- Provide the correct framework within the EU to have competent laboratories for the analysis of CTXs in food.

Risk Assessment:

- Systematically record possible cases of CFP with strict identification of symptoms and nature and origin of suspicious food.
- Structural and toxicological characterization of CTXs and other toxins present in food and microalgae associated with CFP. Pursue recognition of CTX presence in *Seriola* spp. in the NE-Atlantic and consider extending analysis to other fish located in areas with presence of *Gambierdiscus* spp.
- Follow benthic microalgal distribution of hazardous species with special focus on *Gambierdiscus* spp.
- Evaluate exposure to CTXs.

Risk Management:

- Revise current legislation on CFP and foresee the set-up of expert analysis groups to identify deficiencies and future needs.
- Establish preliminary monitoring programs in areas where CFP is present.
- Centralize records of CFP cases in Europe for epidemiology surveillance.
- Establish CFP treatment protocol(s).
- Work in association with food import companies, local fisheries agencies in ciguatera areas and tourism agencies to establish action and communication protocols.

Risk communication

- Define a protocol for a widespread communication strategy to the general public to be used in case of a ciguatera episode.
- Define and implement selective communication targeted at agencies and specific bodies.

## 5. Conclusions

CFP is managed according to national or regional strategies, often dependent on the regional characteristics of CFP, *i.e.*, toxin profiles, implicated fish species, local food habits, *etc*. However, there is also a lack of consensus world-wide on several CFP issues, especially regarding the methods for CTX determination in food. Several factors may be responsible for the present situation: (i) lack of certified standards and reference material, (ii) lack of a coordinated action among laboratories, and (iii) frame of applicability of the method (rapid screening *versus* accurate and confirmatory methods). A worldwide coordinated action could certainly improve adopting harmonized strategies to face-up to CFP and especially favor the establishment of a validated reference method for CTX determination in food. Such a method could be be used for unequivocal identification and accurate quantification of CTXs and as reference for the validation of more rapid screening methods. To achieve this objective, obtaining certified standards and reference material, exchanging samples and setting-up inter-laboratories comparative studies is needed. Possibly, LC-MS/MS is the most probable candidate to become the reference method for CTXs once certified standards are available, a standard operation procedure is validated, and the required limits of detection are guaranteed. Additionally, functional assays such as CBA or RBA have arisen as promising techniques for high-throughput screening of samples and therefore efforts should be also carried out to validate them.

This coordinated action could also consider a common risk analysis strategy to improve risk assessment, risk management and risk communication, in order to reduce the impact of CFP worldwide. If one may expect that this rationale would be shared by the overall scientific community and managers dealing with CFP, one may conclude that the present key issue would be to identify the platform or forum that could lead this coordinated action. Certainly all co-authors herein would favor international cooperation in this matter.

CFP, mainly distributed in tropical and subtropical areas, is already affecting regions in temperate latitudes such as Europe. As CFP intoxications are being reported in European hospitals, methods for CTX identification are needed to confirm these cases. Once this (these) method(s) are available, statistics and epidemiological records regarding CFP affected people in Europe should also improve.

Field studies should also contribute toward better understanding the impact of CFP in Europe. Understanding the distribution of CFP toxin-producing benthic dinoflagellates and evaluating CFP toxins in fish and microalgae should allow a better prediction of CFP risk. Additionally, this information would contribute to hypotheses regarding the possible expansion of CFP towards northern latitudes as a consequence of climate change. Improving detection of CTXs and understanding CFP spatial distribution will contribute to improve the risk analysis of CFP in the European geographical area.

## Figures and Tables

**Table 1:** Diversity of CTXs isolated from fish and microalgal origin [92,94–100].

Origin		Number of rings	Number of carbons	Examples of CTX	Molecular Weigh	Source
**Pacific (P-)**	**Type I**	13	60	CTX (CTX1B, CTX-1)	1110.6	carnivorous fish
				CTX2A2 (CTX-2, 52-epi-54-deoxyCTX)	1094.5	carnivorous fish
				CTX2B2 (CTX-3, 54-deoxyCTX)	1094.5	carnivorous fish
				CTX4A	1060.8	*G. toxicus*, *G. polynesiensis*
				CTX4B (GTX-4B, Gt 4b)	1060.8	*G. toxicus*, *G. polynesiensis*, herbivorous fish
	**Type II**	13	57	CTX3C	1022.8	*G. toxicus*, *G. polynesiensis*, herbivorous fish
				CTX2A1 (2,3-dihydroxyCTX3C)	1056.0	carnivorous fish
**Caribbean (C-)**		14	62	CTX-1	1140.7	carnivorous fish
				CTX-2	1140.7	carnivorous fish
**Indian (I-)**		nd	nd	CTX-1	11406	carnivorous fish
				CTX-2	1140.6	carnivorous fish
				CTX-3	1157.6	carnivorous fish
				CTX-4	1157.6	carnivorous fish

**Table 2:** Principal ciguatoxin congeners analyzed by HPLC-MS with their corresponding molecular ions masses.

Name	Alternative name	Molecular ion [M + H]^+^	Source	References
**Pacific ciguatoxins**				
**P-CTX-1**	CTX1B	1111.6	Carnivorous fish	[45,271]
**P-CTX-2**	CTX2A2; 52-epi-54-deoxyCTX	1095.5	Carnivorous fish	[45,271,274]
**P-CTX-3**	CTX2B2; 54-deoxyCTX	1095.5	Carnivorous fish	[45,271]
**49-*epi*-CTX-3C**	CTX-3B	1023.6	*G. toxicus*	[69,99]
**M-seco-CTX-3C**		1041.6	*G. toxicus*	[69,99]
**CTX-3C**		1023.6	*G. toxicus*	[99,122]
**2,3-dihydroxyCTX-3C**	CTX-2A1	1057.6	Carnivorous fish/*G. toxicus*	[197,122]
**51-hydroxyCTX-3C**	CTX-2C1	1039.5	Carnivorous fish	[197]
**CTX-4B**	GT-4B	1061.6	*G. toxicus* Herbivorous fish	[29,122,227]
**52-*epi*-ciguatoxin-4B**	CTX-4A; GT-4A	1061.6	*G. toxicus*	[100,122,227]
**Caribbean ciguatoxins**				
**C-CTX-1**		1141.6	Carnivorous fish	[94,97,98,102,147,272]
**C-CTX-2**	56-*epi*-C-CTX-1	1141.6	Carnivorous fish	[94,97,98,102,147]
**C-CTX-1127**		1127.6	Carnivorous fish	[97,98,147]
**C-CTX-1143**		1143.6	Carnivorous fish	[97,98,147]
**C-CTX-1157**		1157.6	Carnivorous fish	[97,98,147]
**C-CTX-1159**		1159.6	Carnivorous fish	[97,98,147]
**Indian ciguatoxins**				
**I-CTX-1**		1141.6	Carnivorous fish	[96]
**I-CTX-2**		1141.6	Carnivorous fish	[95,96]
**I-CTX-3**		1157.6	Carnivorous fish	[95,96]
**I-CTX-4**		1157.6	Carnivorous fish	[95,96]

**Table 3:** CFP risk assessment. A synthetic approach for the identification of risk factors.

Level of information	Factors to be addressed	Impact and application (examples) within and beyond risk assessment
**Food intoxication and social aspects**		
**Food**	Toxin content, unequivocal species identification, origin of food, traceability	Species based risk association, hazard characterization, food retrievals, elaboration of safe lists, identification of irregular practices, fraud.
**Symptoms**	General, gastrointestinal, cardiovascular, neurological, time lapses, duration, intensity	CFP diagnosis, regional characterization
**Epidemiology**	Clinical records, restaurant records, intoxication surveys, unreported cases, treatment	Risk characterization, populations at risk, therapy assessment, alternative food sources, epidemiological survey, databases
**Food habits, market**	Consumer surveys, fish trade and sales, fishing practices	Eating habits, exposure assessment, identify areas at risk
**Ethnology**	Avoidances, remedies	Historical learning, local palliative measures, identify population exposed
**Environment**		
**Environmental data**	Latitude, temperature, salinity, turbulence, turbidity, currents, stratification	Microalgal distribution, ecophysiology, toxin production, fish distribution, fish migration, prediction of potential expansion
**Habitat**	Benthic structure, benthic communities, coral status (bleaching), anthropogenic activities	Surface availability for *Gambierdiscus* growth, population succession
**Toxins**	Chemical structure, toxicological potency, toxicological factor for each CTX derivative	Toxin characterization, hazard prediction according to toxin analysis, regional differences according to toxin profiles
**Microalgae**	*Gambierdiscus* spp., other benthic dinoflagellates and microalgal species producing toxins, species distribution and population dynamics, toxin content of natural populations and of clone cultures	Identification of causative toxin producer species, comparative approach among sites, identification of hot spots, temporal variations, intra-specific variation, definition of monitoring strategies
**Fish**	Taxonomy, toxin content, transmission, bioaccumulation and metabolization. Toxins: intra-specific variation/organs/age-size. Trophic level: herbivorous, carnivorous. Behavior: Migration	Species identification, risk associated to species, age, size Species distribution Toxic potency, identification of indicative species

**Table 4:** CFP risk management. A synthetic approach for the identification of key levels where action may improve CFP risk assessment and reduce the risk and impact of CFP in a given area.

Level of action	Factors to be addressed	Impact and application (examples)
**Coordination between implicated agencies**	Define agency to coordinate actions on CFP at regional level Define a common strategy for the management of CFP Centralization of the information and multi-lateral communication Define competent authorities Identify toxins addressed, including derivatives	Optimizes reaction and preventive actions Reduces the impact and cost of CFP
**Legislation**	Define acceptable levels of toxins in food Official methods for toxin recognition CFP ranking of species or groups of fish to be avoided or controlled Responsibilities of fishermen and retailers Import/export policies	Provides a framework for strategy and decision making and defines responsibilities
**Laboratory**	CTX standards Reference material Methodology set up and standardization	CTX identification and quantification
**Monitoring**	Toxin content in fish with validated methods Fish identification *Gambierdiscus* spp. population dynamics Toxin content in *Gambierdiscus* spp. with validated methods Environmental data (e.g., seawater temperature)	Optimizes the strategy for toxin recognition in food and the identification of fish and areas at risk Reduces CFP records Relation between onset of ciguatera and climate change
**CFP records**	Symptom diagnosis Confirmation of CTX in blood/plasma of patients Regional-based epidemiological data	Provides morbidity statistics Characterizes specific regional symptoms Reduce misidentification of other types of non ciguatera ichtyosarcotoxisms
**CFP treatment**	Medical board awareness on CFP Definition of treatment protocols Availabiliity of treatments	Reduces personal CFP symptoms Reduces hospitalization and economic impact
**Industry/Market/Consumer**	Define alternative food sources and avoidable bad practices Define own strategies to overcome CFP according to regional recommendations Report CFP intoxications Traceability of processed food	Reduce CFP records Reduce CFP impact on health and industry (fisheries and tourism)

**Table 5:** CFP risk communication.

Level of communication	Factors to be addressed	Impact and application (examples)
**Widespread**	Listing of species and areas at risk Listing of CFP symptoms Define consumers actions to take in case of CFP Report CFP episodes Report ciguatera warnings and specific instructions during episodes Evaluate public perception and knowledge on ciguatera	Avoidance of fishing and marketing selected species of fish Avoidance of viscera consumption Favor CFP reporting by consumers Improve risk communication strategies
**Selective target**	Facilitate communication and training of administration staff, retailers, fishermen, travel agencies and consumers Listing of CFP symptoms to favor CFP reporting by medical staff Disseminate protocols of communication among agencies Facilitate transfer of information to research agencies involved in the study of ciguatera Evaluate professional perception and knowledge on ciguatera	Reduce CFP impact Improve CFP epidemiological records Understand efficiency of CFP therapies Improve scientific comprehension of ciguatera Improve risk communication strategies
